# Full-Process Detection Technologies for Micro-LED Advanced Manufacture

**DOI:** 10.34133/research.1214

**Published:** 2026-03-31

**Authors:** Hao Su, Junlong Li, Wei Huang, Shuqian Zhang, Jiawen Qiu, Kun Wang, Yongai Zhang, Xiongtu Zhou, Tailiang Guo, Chaoxing Wu

**Affiliations:** ^1^ School of Physics and Information Engineering, Fuzhou University, Fuzhou 350000, People’s Republic of China.; ^2^ Fujian Science and Technology Innovation Laboratory for Optoelectronic Information of China, Fuzhou 350108, People’s Republic of China.

## Abstract

With the continuous advancement of micro-light-emitting diode (Micro-LED) manufacturing technology, Micro-LED displays are poised to emerge as the next-generation display technology, making important strides in areas such as near-eye displays, large-scale high-definition devices, and flexible screens. Among the various technical challenges associated with Micro-LED displays, detection plays a crucial role in identifying defective pixels, improving yield, and reducing overall production costs. Given that Micro-LED chip arrays often consist of millions of tiny chips (<50 μm), existing electrical detection methods face limitations such as low efficiency and high costs. As a result, enhancing detection efficiency, improving accuracy, and reducing costs have become the key trends in the development of Micro-LED detection technology. This review first outlines the key indicators to be monitored during Micro-LED chip detection. It then provides an in-depth analysis of the various detection methods available at different stages of the manufacturing process. Finally, it concludes with a summary of the current state of Micro-LED detection technology and offers a forward-looking perspective on future technological advancements.

## Introduction

Micro-light-emitting diodes (Micro-LEDs) refer to LEDs with dimensions smaller than 50 μm. Due to the use of inorganic materials, primarily GaN and its derivatives, Micro-LEDs offer numerous advantages, including low power consumption, long lifespan, compact size, and high reliability. These characteristics position Micro-LEDs as a next-generation light-emitting technology for applications in optical communication, solid-state lighting, and flat-panel display technologies [1–8]. With the ongoing advancements in process technology, Micro-LED displays have shown significant promise in areas such as near-eye and flexible displays [9–16], making them a highly anticipated technology with vast market potential, often regarded as the “ultimate display technology”.

The origin of Micro-LED dates back to 2000 when Jin et al. [17] introduced a Micro-LED chip with a diameter of approximately 12 μm. Through electrical and optical testing, they demonstrated that the quantum efficiency of these Micro-LEDs surpassed that of traditional-sized LEDs, marking a crucial milestone in the development of Micro-LED displays. Over the following decades, Micro-LEDs gradually gained traction in high-end display devices. Notable milestones include Sony’s 55-inch “crystal LED display” showcased at the 2012 Consumer Electronics Show (CES), using new self-luminous display technology. In 2018, Samsung revealed the world’s first 146-inch modular Micro-LED TV, called “The Wall”, at CES. Apple followed suit at the 2019 Worldwide Developers Conference (WWDC), unveiling its Pro Display XDR, which not only demonstrated Apple’s technological leadership but also pioneered high dynamic range (HDR) workflows in its ecosystem, setting a new benchmark for professional displays. In addition, display manufacturers such as PlayNitride, LG, Vistar, and BOE are establishing Micro-LED production lines. However, despite the growing promise of Micro-LED technology, most devices remain in the experimental or laboratory stage, largely due to their high manufacturing costs. Detection, as one of the critical steps in Micro-LED production, plays a pivotal role in improving efficiency and reducing costs, ultimately helping to lower the overall cost of Micro-LED displays. The quality of Micro-LED chips directly influences the performance of Micro-LED displays. However, the current production processes cannot ensure that all chips meet the required optical and electrical standards. Given the strict yield requirements for Micro-LED devices, comprehensive detection throughout the entire production process is essential. Before mass transfer, detecting and eliminating defective Micro-LED chips at the full process becomes crucial in improving yield.

**Fig. 1.**
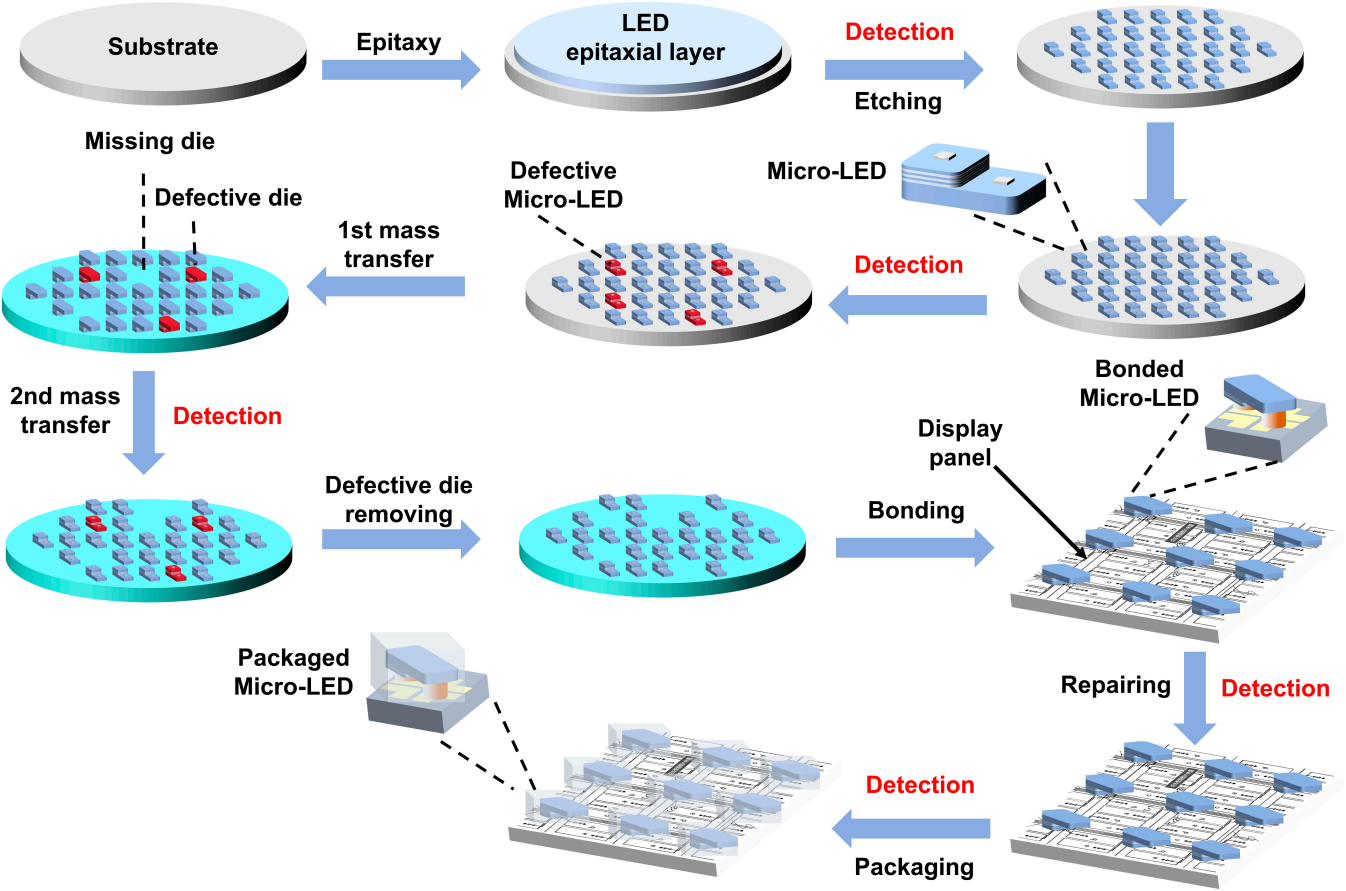
The manufacture process of Micro-LED.

Full-process detection is essential in the Micro-LED manufacturing. As illustrated in Fig. 1, the Micro-LED production process involves several stages: LED epi-wafer, chip on wafer (COW), chip on carrier (COC) and packaging, and the panel stage. Large-scale displays such as 8K screens require around 90 million Micro-LED chips. This detection process must be performed on all chips, posing a significant challenge for current detection methods. Moreover, the high density of Micro-LED arrays results in significant crosstalk between individual pixels. In addition, the specific surface area of Micro-LEDs significantly increases compared to LEDs, and the impact of sidewall defects on the performance of Micro-LEDs greatly increases. Therefore, existing detection technologies struggle to simultaneously satisfy the core requirements of low missed detection rates, short detection times, high-precision detecting, and nondestructive detecting. To address the technical difficulties, researchers have developed a series of detection technologies that include optical, electrical, and microstructural characterization. Therefore, conducting systematic collation and summary of existing Micro-LED detection technologies can provide clear technical references and directional guidance for researchers in relevant fields. This review first outlines key parameters that need to be monitored during Micro-LED detection. It then provides a detailed examination of existing or proposed detection methods at different stages. Finally, it concludes by summarizing the state of Micro-LED detection technology and offering insights into future technological developments. This comprehensive review of current Micro-LED detection technologies aims to provide new ideas for professionals in the field and offer theoretical and practical guidance for advancing Micro-LED detection technologies and their industrial applications.

## Micro-LED Detection Parameters

When it comes to the parameters required for Micro-LED detection, several key characterization metrics are commonly used to evaluate the performance of Micro-LED chips. These include electrical, optical, thermal, and surface morphology parameters, as described in Table 1.

**Table 1.** Important parameters and introduction of Micro-LED

Parameter type	Parameter	Meaning	Figure
Electrical parameters	Forward voltage (*V*_F_)	Referring to the voltage drop across the Micro-LED when it conducts current in the forward direction (i.e., when it is turned on and emitting light).	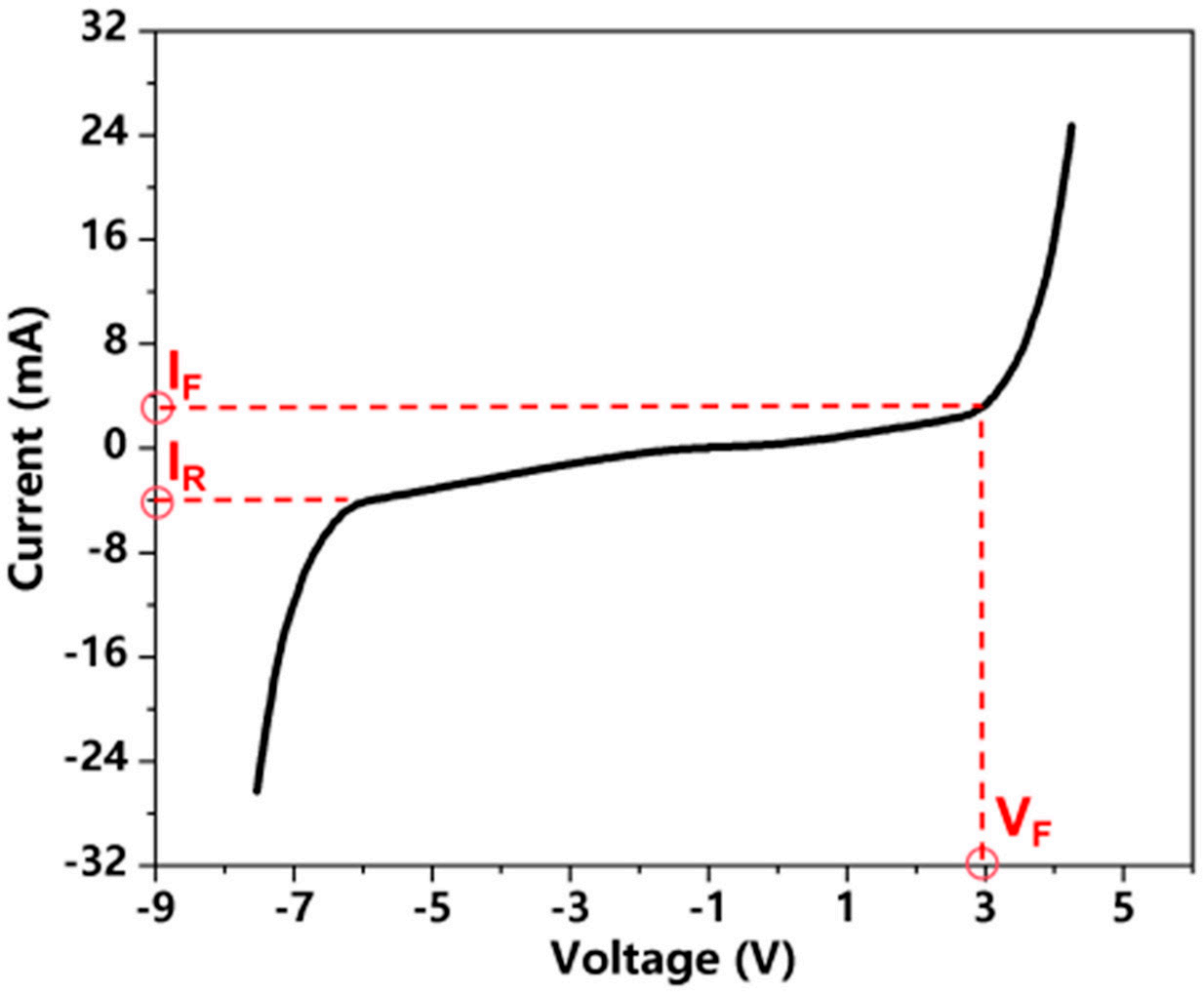
	Forward current (*I*_F_)	Referring to the current flowing from its P-terminal to N-terminal when the LED is forward-biased (i.e., turned on and emitting light).	
	Reverse leakage current (*I*_r_)	Referring to the tiny current flowing from its N-terminal to P-terminal when the Micro-LED is reverse-biased (voltage applied opposite to forward conduction).	
Optical parameters	Peak wavelength (*W*_P_)	Referring to wavelength where luminous intensity is the highest.	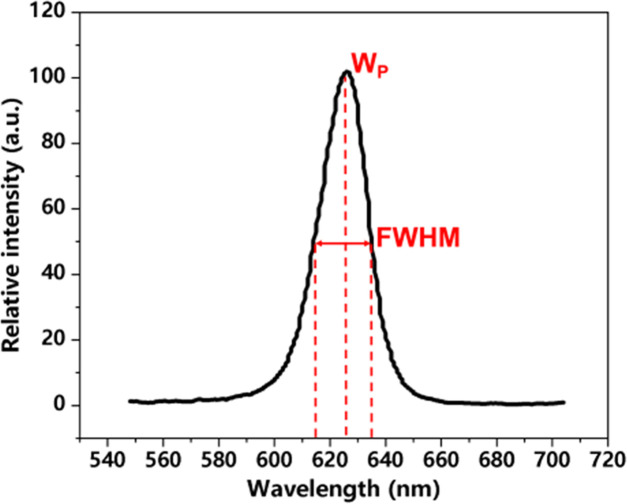
	Full-width at half maximum (FWHM)	Referring to the width of the Micro-LED’s emission spectrum between 2 wavelengths where the luminous intensity drops to half of its peak value.	
Thermal parameters	Junction temperature (*T*_j_)	Referring to the operating temperature at the core light-emitting region of a Micro-LED. It is a pivotal parameter because the ultra-small, densely packed pixels of Micro-LEDs make heat dissipation challenging.	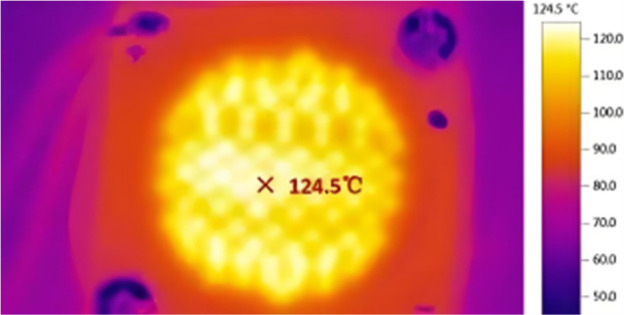
Appearance parameters	Surface morphology	Referring to the contamination or damage on the surface morphology and physical positional deviations of Micro-LED, including electrode absence, completely flipped, lateral flipped, rotational displacement, planar displacement, contamination, and crack.	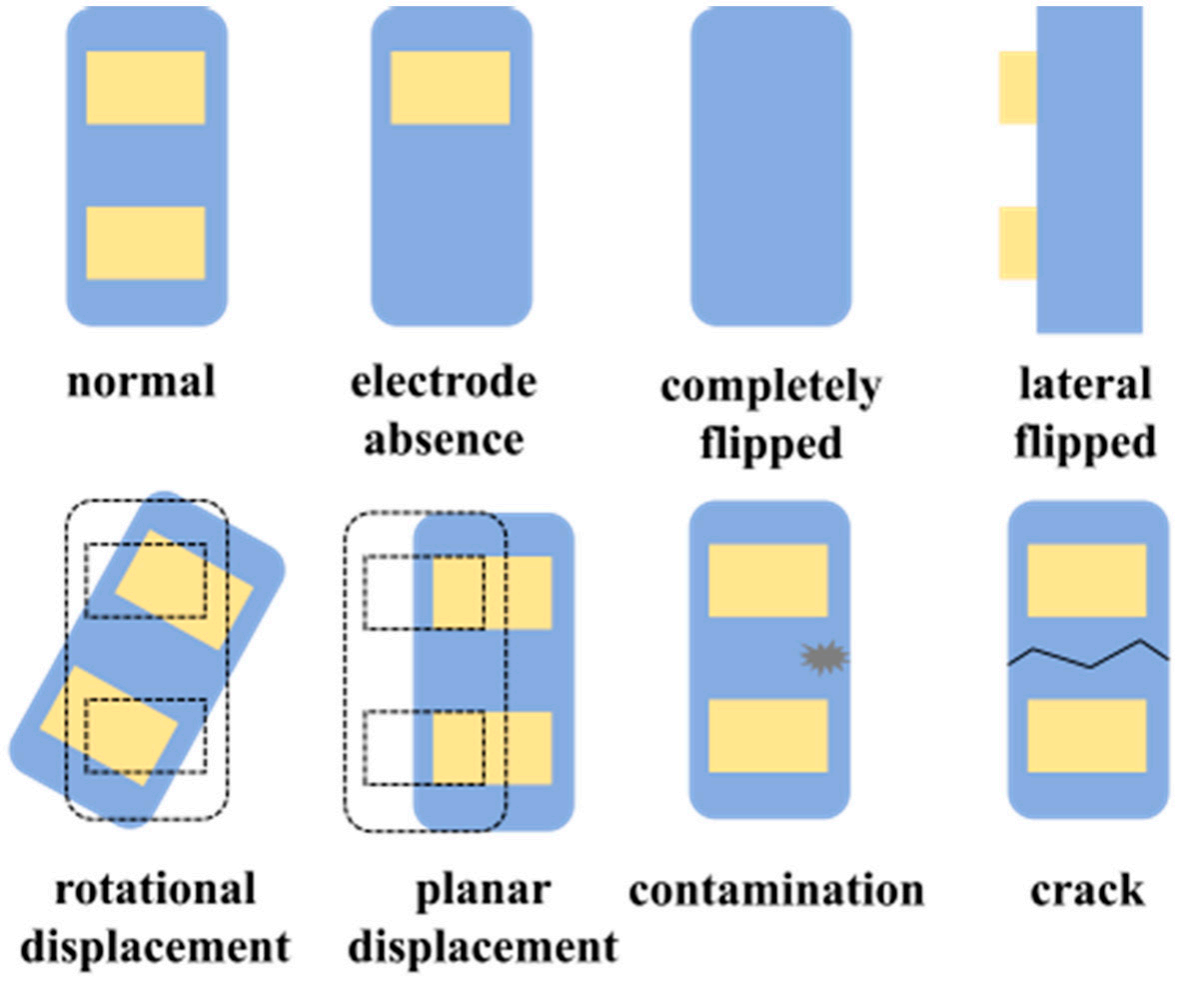

Electrical parameters typically include forward voltage (*V*_F_), forward current (*I*_F_), and reverse leakage current (*I*_r_). *V*_F_ is the minimum voltage required for the Micro-LED to turn on and emit light. The increase in *V*_F_ means that the ohmic contact degrades, the series resistance increases, and the overall power consumption increases. The decrease in *V*_F_ means that parallel leakage channels appear inside the material, causing injection current to be diverted and electro-optical conversion efficiency to decrease, which is usually caused by sidewall defects. *I*_F_ is the operating current applied under this forward voltage, which directly determines the luminous intensity and power consumption. *I*_r_ refers to the very small, undesirable current that flows when a reverse voltage is applied to the non-operating LED, indicating junction quality. The increase in *I*_r_ is often related to the increase in sidewall defects. The sidewall defects introduced by etching form defect states, which become leakage channels and recombination centers for charge carriers. Under high pressure or high temperature stress, defects may cause tunneling or even breakdown.

Optical parameters include peak wavelength (*W*_P_), full-width at half maximum (FWHM), and light intensity. *W*_P_ is the specific wavelength at which the LED emits its maximum optical power, defining its dominant color. FWHM measures the spectral purity by quantifying the wavelength range over which the emission intensity is at least half of its peak value.

Thermal parameters primarily concern the temperature of the PN junction (*T*_j_) when Micro-LEDs are continuously operated for extended periods. The reliability and stability of Micro-LEDs are significantly influenced by *T*_j_. A high *T*_j_ critically affects performance, leading to efficiency droop, color shift, and reduced lifespan, making advanced thermal management essential for reliability.

Regarding surface morphology, the focus is on detecting chip contamination, surface flatness, chip dimensions, chip damage, solder joint defects, and other surface-related imperfections.

In addition to these important parameters, capacitance–voltage (*C*–*V*) characterization is also crucial for Micro-LEDs. It is an electrical characterization method for detecting the internal physical state of a device by measuring its capacitance variation with an applied bias voltage. The core principle lies in the fact that the depletion region width of a PN junction or quantum well (QW) structure changes with voltage, thereby altering its equivalent capacitance. By analyzing the *C*–*V* curve, the doping concentration distribution of the epitaxial material, the built-in potential, and the defect state density at the QW interface can be accurately extracted. This analysis is crucial for evaluating the quality of material growth, understanding the carrier injection and recombination mechanisms, and diagnosing the root causes of efficiency degradation and reliability issues. It provides a key basis for process optimization and performance enhancement of Micro-LEDs.

It should be noted that there is no unified and mandatory specific parameter value standard in the global Micro-LED industry. The main reason for this situation is that the relevant technology of Micro-LED has not yet been solidified, the technology updates and iterates rapidly, and the chip parameters prepared by different manufacturers are different, making it difficult to form a unified standard. An example can be found in the products of Shanghai Tulinopto Technology Co. Ltd. One of their blue products has a size below 50 μm. This product has a *V*_F_ of 2.56 V, an *I*_F_ of 0.5 mA, and an *I*_r_ of 0.2 nA. Their other blue product, with a size of 60 μm, has a *V*_F_ operating range of 2.6 to 3.1 V, an *I*_F_ of 1 mA, and an *I*_r_ range of 0 to 1 μA. It can be seen that even for the same manufacturer, the parameter standards for different products are different, and the definition of Micro-LED is also vague.

In addition to parameters, we discuss the industrial adaptability of Micro-LED detection technology using the following 6 aspects: industry maturity, technical economics, detection efficiency, technical simplicity, detection accuracy, and data comprehensiveness.

**Fig. 2.**
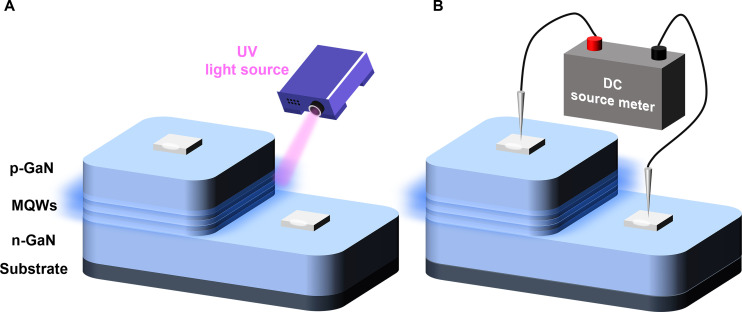
The luminescence principle of (A) PL and (B) EL.

Industry maturity refers to the development maturity of this detection technology in Micro-LED process. The earlier a certain technology appears, the slower its updates and iterations, and the wider its scope of use, the higher the industry maturity of that technology. Technical economics refers to the cost of consumables generated by the detection technology during the detection process. The higher the cost required for a single test, the higher the technical economics. Detection efficiency is used to measure the efficiency of detection technology. The more chips that can be detected simultaneously during a single detection process, the higher the detection efficiency. Technical simplicity refers to the degree of simplification of the equipment used in the detection technology. The simpler the equipment used in the detection process, the higher the technical simplicity. Detection accuracy refers to the accuracy of the data obtained by the detection technology. As we all know, conventional electroluminescence (EL) detection technology boasts the highest accuracy, whereas that of photoluminescence (PL) detection is relatively lower. The fundamental reason is that the 2 detection methods are based on different principles, as shown in Fig. 2. The principle of PL is that photons of short-wave high-energy light waves are directly injected into the light-emitting layer of Micro-LED chips to induce PL. As long as the structure of the light-emitting layer is intact, PL occurs. In contrast, in EL detection, externally injected electrons need to pass through the conductive path of the electrode light-emitting layer electrode to enable Micro-LED to emit light normally. If defects occur in this path, it will cause the Micro-LED chip to malfunction. Therefore, in the actual detection steps, there may be a phenomenon of artificially high yield rate in PL detection (the difference in luminescence principle between the 2 is shown in Fig. 2A and B). Data comprehensiveness refers to the completeness of the data obtained by detection techniques. Due to the detection principle, PL detection technology cannot obtain the electrical parameters of Micro-LEDs. Therefore, in terms of data comprehensiveness, PL detection is inferior to EL detection.

The light emission performance and carrier lifetime of Micro-LED are also important criteria for measuring their performance. Correspondingly, measuring the transmittance/reflectance of epitaxial wafers or COW wafers, observing whether the device structure can emit as much light as possible in the same direction, and using time-resolved PL (TRPL) to observe carrier lifetime are also important steps in Micro-LED detection. In addition, cathodoluminescence (CL) has also been applied in Micro-LED detection. When high-energy electron beams bombard the surface of Micro-LED chips, a large number of secondary electron and hole pairs are excited. These charge carriers will diffuse and recombine within the material, with some of them releasing photons through radiative recombination. However, the focus of these detection methods is to visualize certain specific data of Micro-LED, and then use these data to guide and improve the preparation process of Micro-LED. These detection and analysis methods are not suitable for the Micro-LED manufacturing line, so they will not be introduced in detail in this article.

## LED Detection Methods

Full-process detection is critical for the preparation of Micro-LEDs, requiring quality checks at various stages throughout the entire manufacturing process. Accordingly, this work classifies Micro-LED detection methods based on different process stages. The detection methods can be divided into 3 main categories: EL detection, PL detection, and automatic optical inspection (AOI). In EL detection, 2 probes are used to inject carriers into the Micro-LED, inducing EL. This allows for the measurement of parameters such as the emission spectrum, threshold voltage, and *T*_j_. In contrast, PL detection uses short-wavelength excitation light [such as ultraviolet (UV) light] to inject photons into the Micro-LED. This process causes radiation recombination between electrons and holes in the QWs. AOI, on the other hand, combines high-resolution cameras with deep learning algorithms to analyze the field of view images of Micro-LEDs under background lighting. For an AOI system, a specialized algorithmic model must be developed and trained based on the image characteristics of the target Micro-LED chips. During the detection phase, images of the Micro-LED array are input into the pretrained model, allowing the system to automatically screen and identify defective chips.

**Fig. 3.**
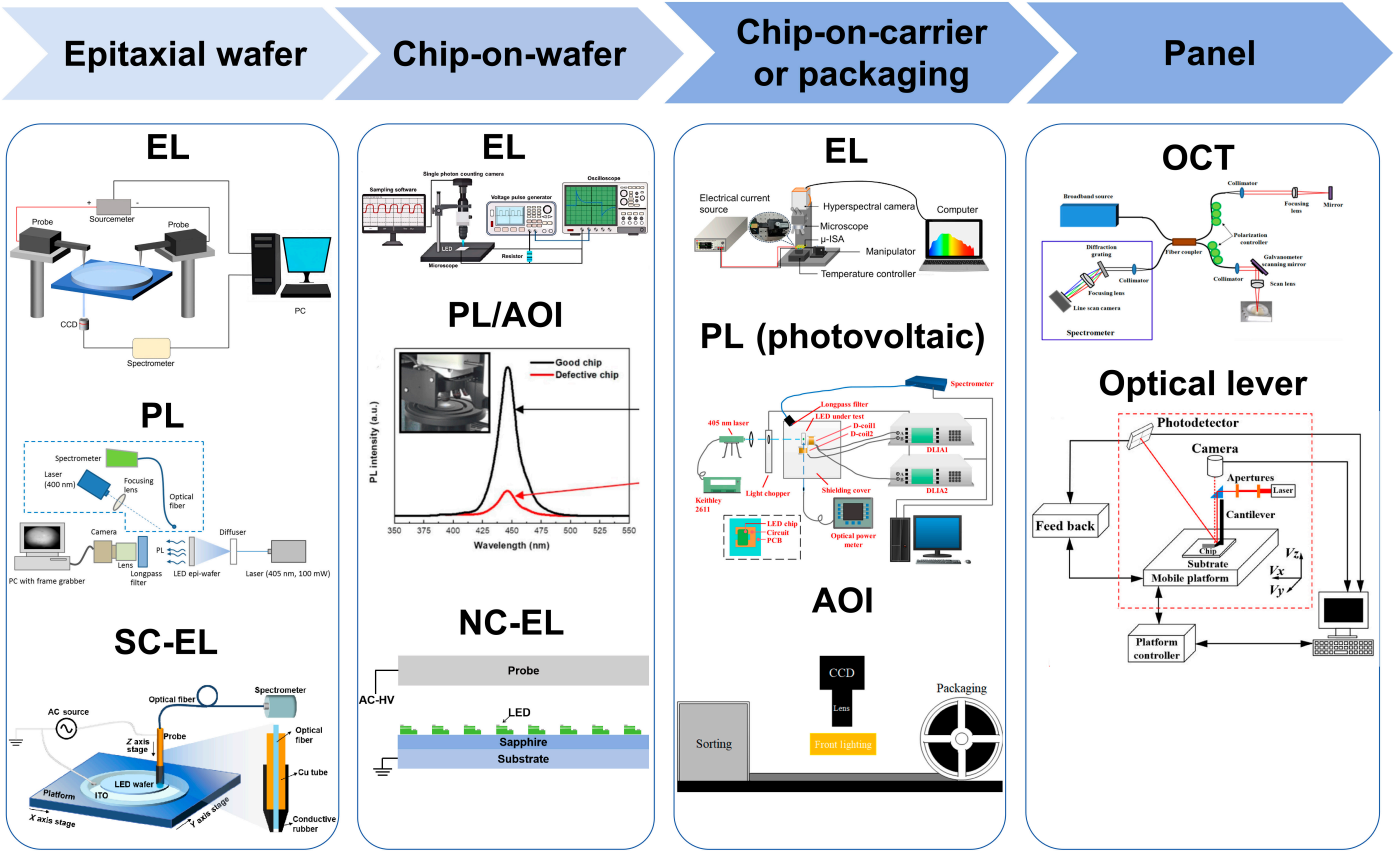
Micro-LED detection methods. Reprinted with permission from [21], under the CC BY 4.0 license. Reprinted with permission from [22], under the CC BY 4.0 license. Reprinted with permission from [41]. Copyright 2025 American Chemical Society. Reprinted with permission from [60], under the CC BY 4.0 license. Reprinted with permission from [85]. Copyright 2023, IEEE. Reprinted with permission from [86]. Copyright 2024, IEEE. Reprinted with permission from [88]. Copyright 2021, Elsevier. Reprinted with permission from [99], under the CC BY 4.0 license. Reprinted with permission from [101]. Copyright 2025, Elsevier.

In conventional manufacturing processes, EL spectroscopy is generally considered more accurate than PL spectroscopy. However, EL detection is more time-consuming. Taking the EL detection in the epi-wafer stage as an example, during EL detection, the edges of the epi-wafer must first be etched until the n-GaN layer is exposed. Then, 2 high-precision probes are placed in contact with the p-GaN and n-GaN layers, respectively, and carriers are injected through the probes. This method not only requires additional time for etching the epi-wafer but also carries the risk of mechanical damage from the rigid probes, which can increase costs. In comparison, PL detection offers much higher detection efficiency than EL detection. However, due to its different luminescent mechanism, the accuracy of PL detection is often regarded as inferior to that of EL detection. AOI is another fast and efficient method for identifying surface structural defects in Micro-LEDs. It enables batch defect detection of thousands of Micro-LED chips in a single image without chip-by-chip detection. Moreover, AOI avoids the excitation steps required for EL/PL, such as the moving electric probes or applying UV light to the sample, thus cutting detection time significantly. However, AOI cannot detect internal defects, and the overall quality of Micro-LEDs must still be validated through EL detection. Although such LEDs employ various types of substrates, only substrates with very low transmittance (e.g., silicon) produce strong reflected light during AOI, which can interfere with the optical system and the algorithm in judging chip quality. The other detection methods discussed can be applied to samples with any type of substrate. Additionally, both EL and PL detection methods are compatible with Micro-LEDs on various substrate types. In contrast, noncontact EL (NC-EL) detection requires coupling a metal probe to the Micro-LED chips during detecting, and this method is only suitable for substrates that are optically transparent from the bottom, such as sapphire. The key detection methods for Micro-LEDs corresponding to different process stages are summarized in Fig. 3. Next, we will introduce the specific detection methods used at each stage of Micro-LED production, including epi-wafer detection, chip-on-wafer (COW) detection, chip-on-carrier (COC) and packaging detection, and panel detection. It should be noted that all LEDs referenced in the detection-related literature cited in this work are GaN-based LEDs.

### Epi-wafer detection

Since GaN-LED chips are produced by etching LED epi-wafers, the quality of the LED epi-wafer plays a crucial role. A high-quality wafer should exhibit uniformity in EL wavelengths and electrical parameters, which is essential for creating high-quality GaN-LED chips [18]. Therefore, selecting LED epi-wafers with EL wavelengths and electrical properties that align with display requirements is critical for achieving high-performance displays with consistent brightness and color uniformity [19–27]. As a result, detecting LED epi-wafers before the etching process is essential.

The traditional detection method for LED epitaxial wafers is usually probe detecting. The optical and electrical parameters obtained in this way are very accurate, but the probe inevitably causes some damage to the epitaxial film. To reduce the damage of probes during detection and the damage caused by probes to LED epi wafers, Kim et al. [19] proposed a double-sided electroluminescent measurement system to protect the probes. The entire measurement system is mainly composed of 2 integrating spheres. Two integrating spheres are placed on the upper and lower sides of the LED chip to collect the emitted light from each side. The probe on the front integrating sphere of the system is equipped with a spring, which can act as a buffer when in contact with the LED epi-wafer. When the probe contacts the electrode of the LED chip, it can avoid excessive stress and damage to the LED chip. In addition, the end of the probe is coated with silicon, which can prevent wear and tear of the probe during contact with the LED electrode and reduce the cost of replacing the probe. The mapping properties of the front and the rear included the peak wavelength, peak intensity, FWHM, and current. Forward voltage and *I*_r_ were measured in the front only.

Due to the certain relationship between the photoelectric parameters of epi-wafers and the photoelectric parameters of individual LED chips after etching, the performance of individual LED chips can be evaluated by detecting the performance of LED epi-wafers, thereby improving detection efficiency. In 2016, Shim et al. [20] proposed an “EL Q-check” system. After etching a portion of the epi-wafer to the n-GaN layer using a laser, indium is deposited on the n-GaN as the negative electrode. Then, they cut a scratch from the surface to the substrate at both ends of the epi-wafer using a precise and controllable diamond knife (thickness 1 μm) to form insulation. Next, a diamond knife is used to cut a scratch with a depth from the surface of the epi-wafer to n-GaN at the midpoint of both ends as a leakage current barrier to restrict the conduction path of charge carriers so that the epi-wafer can emit light normally. The experiment shows that when the injected current density is less than 10 A/cm^2^, the main wavelength of the measured EL spectrum has a high degree of agreement with the main wavelength of the LED chip. This method of evaluating LED chips using the overall optoelectronic performance of LED epi-wafers has good fit at low current density and low voltage, but as the current density and voltage increase, this method is no longer applicable. The reason is that a good ohmic contact was not formed during the deposition of indium on n-GaN, resulting in an increase in contact resistance and a low fitting degree at high current densities. Additionally, this method can also obtain the luminescence spectrum and obtain relatively accurate *I*_r_ current. However, this method cannot screen out defective LED chip bad pixels, and the photoelectric parameters of LEDs prepared in different regions of epitaxial wafers are not completely the same. Therefore, this method has certain limitations as a detection method.

**Fig. 4.**
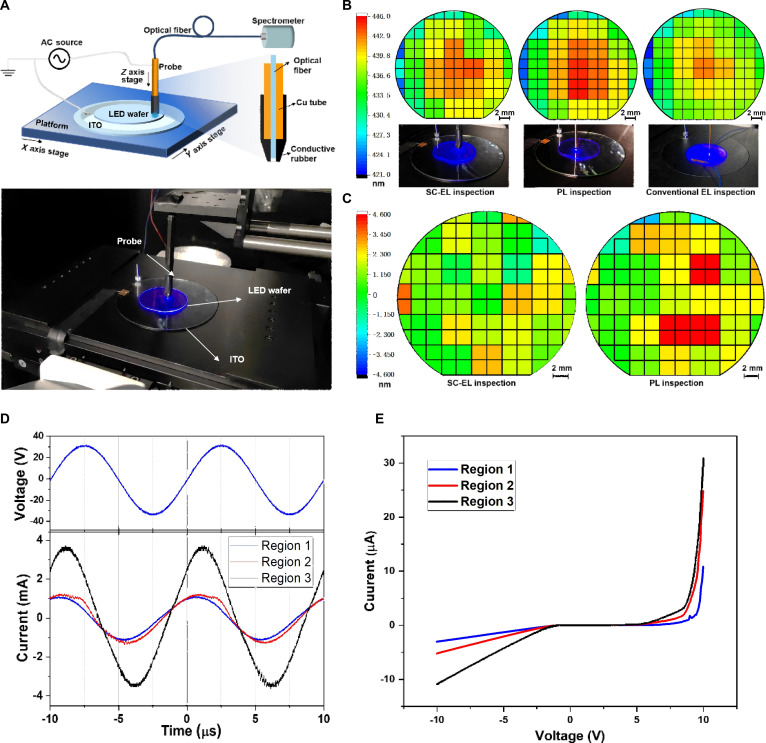
(A) Schematic diagram and physical image of SC-EL detection system [21]. (B) Peak wavelength of luminescence obtained by SC-EL, PL, and conventional EL [21]. (C) Difference between the peak wavelength of luminescence obtained by SC-EL/PL and conventional EL [21]. (D) Waveform of the applied AC voltage (top panel) for SC-EL detection and the measured currents of different LED epitaxial wafer regions [21]. (E) DC *I*–*V* curves of the corresponding LED epitaxial wafer regions [21]. (A to E) Reprinted with permission from [21], under the CC BY 4.0 license.

Recently, the noncontact operation and single-contact operation have been proposed and applied in various light-emitting device structures, including GaN-based LED and quantum dot-based devices. As for the noncontact operation mode, the ohmic contact between electrodes and LED is avoided. Two insulating layers are introduced between the electrodes and the LED. Similarly, for the single-contact operation mode, an insulating layer is introduced between the electrode and the p-GaN terminal (or n-GaN terminal). Due to the presence of the insulating layer, DC voltage cannot drive the LED because the insulating layer would block the injection of charge carriers. Therefore, high-frequency AC power must be used to drive this noncontact or single-contact LED. The advantage of noncontact or single-contact mode is that it does not require complex pretreatment on the LED surface to achieve ohmic contact. This advantage is particularly important in the detection of LED epi-wafers. In 2024, Su et al. [21] propose a nondestructive detection method and system for LED epi-wafers based on soft single-contact EL (SC-EL) operation mode. The schematic diagram of the detection system is shown in Fig. 4A. The proposed SC-EL detection can detect the electrical characteristics as well as the EL properties of LED epi-wafers without introducing mechanical damage and without etching the LED epi-wafers. In addition, it is demonstrated that the SC-EL detection has a higher EL wavelength accuracy than conventional PL detection, as shown in Fig. 4B and C. The superior performance of SC-EL detection is attributed to its differential control over the electrons and holes that drive the luminescence process. In LED epitaxial wafers, electron mobility is higher than that of hole. Consequently, radiative recombination between electrons and holes predominantly occurs within the QWs located near the p-GaN layer. As a result, the EL spectrum primarily reflects the properties of these specific QWs close to the p-GaN side, so does EL detection. In contrast, during PL detection, the recombination of electron-hole pairs takes place across all QWs. Moreover, by using the SC-EL detection, it can also get the electrical characteristics of LED epi-wafers, which cannot be obtained by using conventional PL detection. The experimental results show that there are differences in *I*_r_ in different regions of LED epitaxial wafers, which can be observed from the AC current–voltage (*I*–*V*) characteristic curves obtained by this method, as shown in Fig. 4D and E. The proposed SC-EL detection can ensure high detection accuracy without causing damage to the LED epi-wafer, which holds promising application in LED technology.

**Fig. 5.**
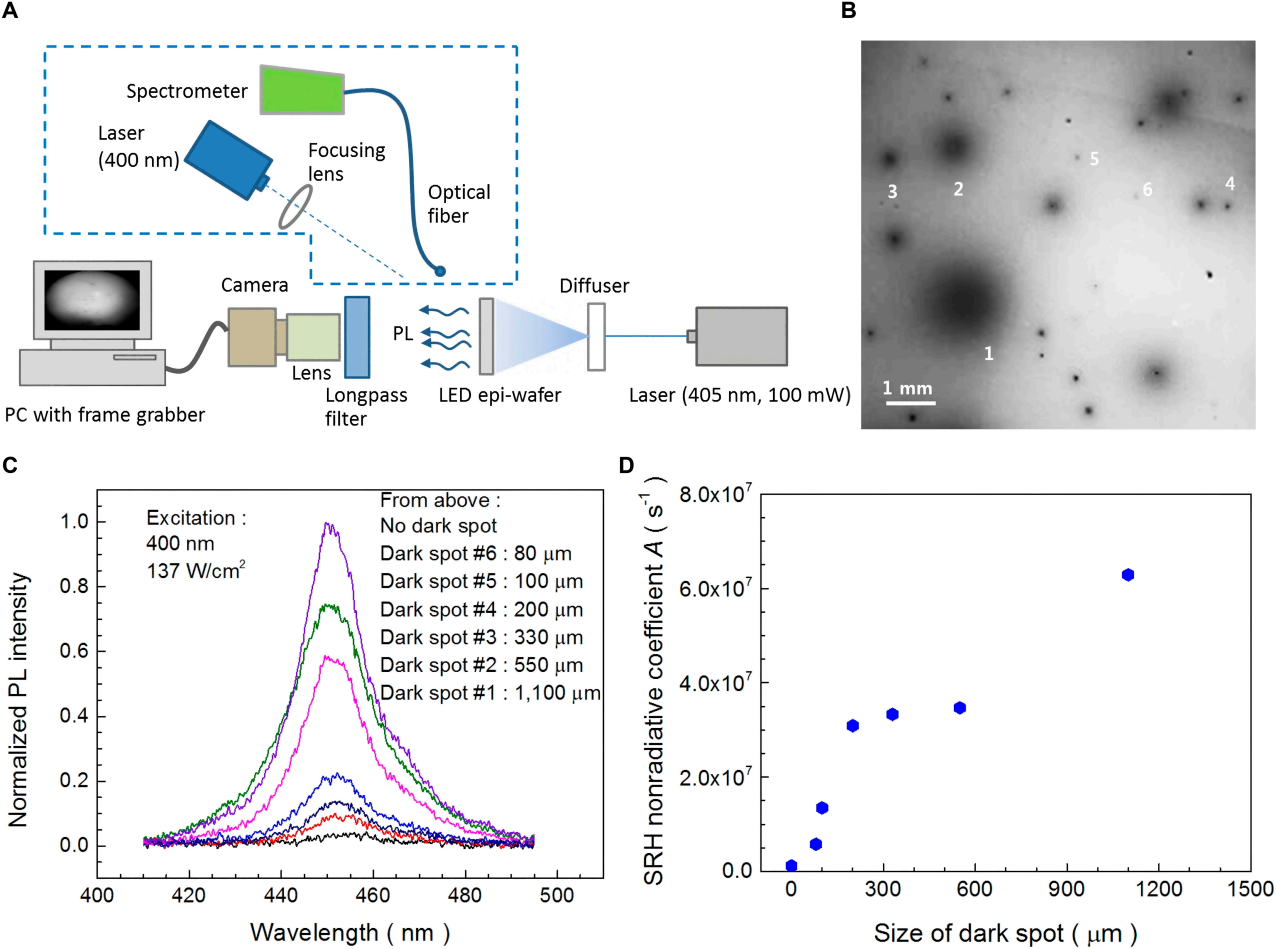
(A) Schematic diagram of the PL imaging setup. The configuration comprised a main system for general imaging, with the components within the dashed-line box specifically employed for PL analysis of selected defective regions [22]. (B) PL images of selected areas with dark spot defect regions of various sizes at a power density of 5 mW/cm^2^ for excitation light [22]. (C) PL spectra obtained from DSDRs of various sizes [22]. (D) SRH nonradiative coefficient as a function of the size of the DSDRs, which was derived by assuming that the radiative recombination coefficient is 1 × 10^−10^ cm^3^/s for the detected regions [22]. (A to D) Reprinted with permission from [22], under the CC BY 4.0 license.

In addition to EL detection, PL detection is also commonly used in epi-wafer detection. The principle is to excite an epi-wafer with short-wavelength excitation light (such as UV light) to produce PL. Obtaining PL images through a camera and analyzing and filtering out defective areas is a commonly used detection method for PL detection. In 2015, Kim et al. [22] proposed a visual camera-based LED epi-wafer PL detection method. The schematic diagram of the detection system is shown in Fig. 5A. A visual camera is used to obtain and analyze PL images of LED epi-wafers and analyzes dark spot defective regions (DSDRs) in the images, as shown in Fig. 5B. They used UV light to irradiate DSDRs of different sizes to obtain PL intensity. The experimental results indicate that the PL intensity decreases with the increase of DSDR size, as shown in Fig. 5C. After that, they assume that the radiative recombination coefficient is 1 × 10^−10^ cm^3^/s to calculate the nonradiative recombination coefficient. The experimental results indicate that the Shockley Read Hall (SRH) nonradiative recombination coefficient increases with the size of the DSDRs, as shown in Fig. 5D. Furthermore, DSDRs were prepared into LEDs, and experimental results showed that DSDRs had a significant impact on both the optical and electrical parameters of LEDs. Under different driving currents, the larger the area proportion of DSDRs in the entire LED chip, the lower the optical power. In addition, the larger the area proportion of DSDRs in the entire LED chip, the greater the required driving current to achieve the same brightness. This detection method is based on visual technology, using a visual camera to analyze the PL images of LED epi-wafers. Due to the high-density nonradiative composite defects of DSDRs, which can affect the normal operation of LED chips, a PL detection method combined with a visual camera is used to quickly evaluate the expected working condition of epi-wafers, thereby estimating the performance of LED chips manufactured from epi-wafers. To adapt to the detection and analysis of Micro-LEDs, it is necessary to further improve the recognition accuracy of the equipment.

### COW wafer detection

COW is mainly fabricated into Micro-LED chip structures on epitaxial layers/sapphire substrates through semiconductor processes such as etching, deposition, and metal evaporation. After multiple process steps, the COW wafer is in a very close state to the Micro-LED packaged on the display panel. Therefore, there are more types of detection methods for the COW wafer stage [28–80]. This article categorizes these detection methods into EL detection, PL detection, AOI, and microscopic defect detection based on the principle of detection.

#### EL detection

EL detection is one of the commonly used detection methods for COW wafer detection. The data obtained by EL detection have the highest accuracy. However, for Micro-LEDs, the drawbacks of EL detection are also evident. Due to the small size of Micro-LEDs (<50 μm), there are millions of Micro-LED chips on the wafer. EL detection requires repeated probe lowering and lifting, which not only consumes a lot of detection time but also may cause mechanical damage to Micro-LEDs, increasing detection costs. Therefore, researchers are trying to improve EL detection from aspects such as efficiency improvement and damage suppression.

The thermal performance of LED determines its efficiency, lifespan, and reliability. Overheating can reduce light efficiency, shorten lifespan, and affect performance, which means that effective thermal management is crucial. However, the electrical and thermal properties of LEDs cannot be detected simultaneously. Compared to conventional sizes, there are more Micro-LEDs on wafers of the same size. Using conventional detection methods is too inefficient for Micro-LEDs. In 2021, Shih et al. [28] proposed a thermocouple probe detecting system that can simultaneously measure the thermal and electrical parameters of Micro-LED. This is a special probe integrated on the printed circuit board (PCB), which is composed of a pair of trapezoid microcantilever probes with a monolithic micromechanical thermocouple. One of the microcantilevers is composed of a single layer of Ni for electrical conduction. Another microcantilever is composed of n-type polycrystalline silicon, SiO_2_, and Ni layers, used for temperature sensing. This pair of micro-cantilevers will undergo elastic deformation upon contact with Micro-LEDs, avoiding stress concentration and reducing mechanical damage to Micro-LEDs. In addition, this probe can simultaneously obtain the electrical and thermal parameters of Micro-LEDs. The results showed that using a 75-μm Micro-LED chip as the detection sample, the temperature of the chip was measured to be 52 °C when a current of 100 μA was applied. Using this method, they successfully measured the correlation between thermocouple voltage and time for Micro-LED chips. Also, they obtained the relationship between driving current and temperature through this method, proving that the method can simultaneously obtain electrical and thermal parameters. This detection method can simultaneously measure the electrical and thermal parameters of Micro-LEDs. The elastically deformable microcantilever also reduces the mechanical damage caused to Micro-LEDs during detection, improves detection efficiency, and reduces detection costs. Considering that the probe is similar to the conventional EL detection method, the position of the probe may deviate from the position of the Micro-LED electrode during the detection process, requiring a reset. In addition, only one chip can be detected at a time and frequent probe lifting and lowering means limited efficiency improvement.

A transparent conductive film that is compatible with the shape of the LED chip electrodes can protect the electrodes of the LED chip from damage during detecting, reducing costs. In 2015, Lin et al. [35] proposed a nondestructive detecting method for LED chips. This method uses a transparent conductive film to cover the LED chip under detect, and the detect needle indirectly contacts the LED chip through the conductive film, thus avoiding the needle mark problem that may occur in traditional needle detecting. The conductive film has transparent conductive grooves, which are divided into positive and negative conductive grooves, corresponding to the positive and negative electrodes of the LED chip, respectively. Its shape and size are consistent with the detected LED chip, and its depth is consistent with the thickness of the detected LED chip electrodes. These specially prepared transparent conductive films are etched on an insulating substrate. During detection, the electrodes of the LED chip are embedded in the transparent conductive grooves, and the detecting needle is inserted into the areas in contact with the positive and negative electrodes of the LED for detecting. This scheme has a simple principle and good effect, but like many methods that use media to separate probes from LED chips and Micro-LED chips, one specification of conductive film can only adapt to one specification of chip, with low applicability. Moreover, as the size of LED chips becomes smaller, the difficulty of preparing conductive media that are compatible with them will also increase.

Probe cards can also be used for COW wafer detecting. This method can simultaneously detect the advantages and disadvantages of Micro-LED chips in the same column or row. Nowadays, some companies have mass-produced this probe card and sold it to the outside world (such as NANO EX). As early as 2015, Du [36] proposed a detection method that can simultaneously detect multiple LED chips. In this method, there are detect pads on both electrodes of each LED chip, and then a transparent probe card is used to contact the detect pads of the LED chip. By applying voltage to the common terminal of the transparent probe card, multiple LED chips can be detected simultaneously. This method of using row and column electrode gating to light up LED chips can effectively improve LED detection efficiency, but only transparent probe cards with contact arrangements that fully correspond to the chip arrangement on the detected LED wafer can be used during detection, which has low applicability. As the size of Micro-LEDs becomes smaller, the difficulty and cost of producing transparent probe cards and detect pads used in this method will increase.

In 2025, Wu et al. [37] proposed a 3-dimensional (3D) flexible probe. They constructed a wafer-level microscale LED high-throughput EL measurement system with the flexible probe as the core. This probe has a similar structure to the rigid probe card used in semiconductor detecting, but it is composed of elastic micro-columns and conductive structures as a whole. The stress generated by this probe when in contact with microscale LEDs is only 0.91 MPa, which is at least 2 orders of magnitude lower than conventional probes. Moreover, due to its elasticity, deformation occurs when in contact with the chip, and the 2 micro-columns can naturally form a height difference during detecting. The probe includes 32 × 32 pairs of elastic micro-columns, which can simultaneously detect 1,024 microscale LEDs within 0.5 s. At the same time, the probe can detect 1 million times repeatedly, greatly reducing detecting costs. However, like many “probe card” schemes, this method is only applicable to specific arrangements of Micro-LED wafers and has low universality. Moreover, in the actual detecting process, testers cannot guarantee that the stress on each pin of the probe card when in contact with the Micro-LED chip is completely consistent. Different stresses will inevitably lead to different injection current sizes, affecting the actual detecting results.

**Fig. 6.**
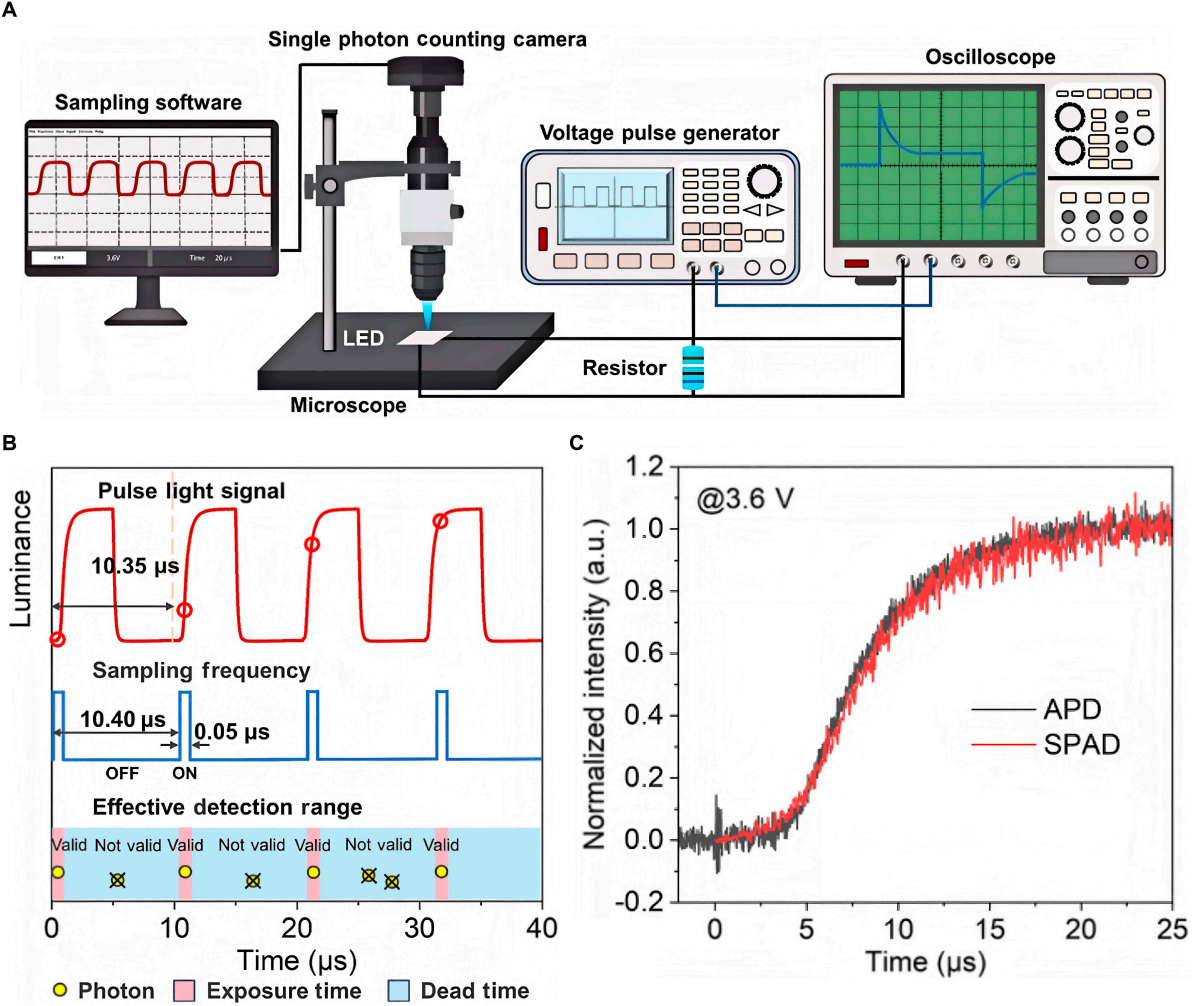
(A) Schematic diagram depicting the TSR-EL system setup, which includes a voltage pulse generator, an oscilloscope, a microscope, and an SPC [41]. (B) Principle of the time-gated sampling method [41]. (C) Comparison of the TREL and TSR-EL curves from a typical organic LED (OLED) device, acquired using an APD and an SPAD detector, respectively, under an applied bias of 3.6 V [41]. (A to C) Reprinted with permission from [41]. Copyright 2025, American Chemical Society.

The typical time-resolved EL (TREL) detection device typically consists of a voltage pulse generator, an oscilloscope, and a photodetector [avalanche photodiode (APD) or photomultiplier tube (PMT)] [40]. However, due to the limitations of APD and PMT detection capabilities, traditional TREL devices are unable to recognize weak signals with low brightness (below 300 cd/m^2^). In addition, APD and PMT are single-pixel detectors without spatial resolution, and there is currently a lack of single-pixel detection methods that can obtain the surface light output of Micro-LED chips at a spatial scale. In 2024, Cao et al. [41] proposed a time-resolved EL spectroscopy (TSR-EL) for characterizing Micro-LEDs. The schematic diagram of the detection system is shown in Fig. 6A. By combining a single-photon camera (SPC) with a time-gated sampling method, they derived the temporal and spatial resolved EL intensity that increases with time. The principle of the time-gated sampling method is shown in Fig. 6B. Because of the high sensitivity of SPC, this method can detect ultra-low EL of devices operating near the conduction voltage during the delay phase. Through this detection method, they first obtained the spatial light distribution of quantum dot LEDs (QLEDs) under different applied voltages and temperatures. The TREL curve of a typical QLED device using an APD detector and the TSR-EL curve derived from the same device using a single-photon avalanche diode (SPAD) detector under 3.6 V is shown in Fig. 6C. In addition, they also obtained temporal and spatial resolution EL information, which proves the high versatility of the detection method. This method can detect and analyze the surface light emission of many types of light-emitting devices with single-pixel accuracy and is suitable for low-brightness micro-light-emitting devices.

**Fig. 7.**
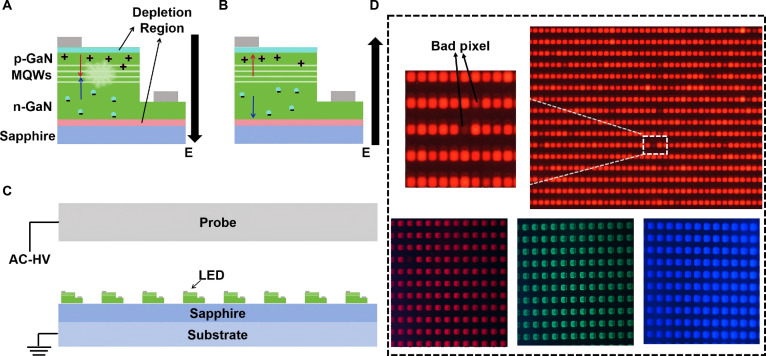
(A) Schematic illustrating the working mechanism of Micro-LED under the forward electric field. (B) Schematic illustrating the working mechanism of Micro-LED under the reverse electric field. (C) Schematic diagram of Micro-LED detection based on non-electrical contact operation. (D) Luminescence image of Micro-LED array.

To avoid mechanical damage to Micro-LEDs during detecting, noncontact operation is the best choice. In 2015, Guo and Wu from Fuzhou University proposed NC-EL detection technology for wafer level Micro-LED chips [43–55]. This technology can perform scanning and NC-EL detection on Micro-LED chips on wafers with millions of orders of magnitude. The theory of NC-EL detection is the most efficient and accurate detection method. This technology can avoid the problem of false high yield caused by PL and does not require precise contact between the probe and the chip, making the detection more efficient. The principle of NC-EL is as follows: When an external electric field is directed from p-GaN to n-GaN, electrons in the n-GaN region and holes in the p-GaN region diffuse and move toward the direction of multiple QWs (MQWs), where radiation recombination occurs, as shown in Fig. 7A. Under forward bias, radiation recombination cannot continue to occur, which means that only one emission can be observed when a DC voltage is applied or during the positive half cycle of AC driving. This is because the drift of most charge carriers will form depletion regions at both ends of the Micro-LED, generating an induced electric field to shield the external electric field and prevent carrier diffusion. Therefore, a reverse electric field must be applied to drive the electrons and holes back to their initial state, as shown in Fig. 7B. Therefore, this detection technology requires the application of an alternating electric field to make the Micro-LED to be detected emit light periodically. The NC-EL of LED can be described by the following equation [48]:

(1) $$i=A[\sin\left(\mathrm{\omega t}+\alpha )\right]\;e^{\frac{{\mathrm{qU}}_{0}}{\mathrm{kT}}\sin\omega t}$$

where *i* is the current during the operation of the entire NC-EL system. *A* is a constant determined by the PN junction reverse leakage current, applied voltage, and insulation layer capacitance. ω is the angular frequency of the applied voltage. *t* is the operating time of the entire NC-EL system. sinα is a constant determined by the PN junction reverse leakage current, thermal voltage, applied voltage, and insulation layer capacitance. *q* is the electron charge. *k* is the Boltzmann constant. *U*_0_ is the amplitude of the applied voltage, and *T* is the thermodynamic temperature. It should be noted that this formula only applies to the working state of noncontact systems after a sufficient amount of time.

The luminescence mechanisms of DC- and AC-driven Micro-LEDs differ fundamentally. Under DC drive, a constant directional voltage enables the continuous injection of charge carriers into the QW region, resulting in stable and sustained radiative recombination luminescence. In contrast, AC drive operates under a periodic alternating voltage, where carrier injection and recombination occur only during the forward-bias half-cycle, while light emission ceases during the reverse-bias or zero-crossing phases. Thus, AC-driven luminescence is inherently intermittent and pulsed. Furthermore, the reverse-bias phase in AC drive facilitates the extraction of some carriers, which may influence the device’s dynamic response. The different working principles inevitably lead to different parameters obtained. However, existing literature suggests that this difference is very small. Taking luminescence spectra as an example, the AC-driven EL spectra are even more accurate than PL spectra [21]. Secondly, for noncontact detection based on the core principle of AC drive, this difference has no impact on the yield of detection products. Because the essence of yield detection is to screen out chips that are too bright or too dark, noncontact detection perfectly fits this detecting scheme. In the process of NC-EL detection, an electrode field plate is used to couple with the LED chip, as shown in Fig. 7C. An external high-frequency power supply is applied to the electrodes of the field board and the bottom electrode of the LED chip array, causing the LED chip array below the field board to emit light simultaneously. Images are collected by a camera, and the brightness parameters of the LED chips are detected. The schematic diagram and actual effect diagram of the detected Micro-LED wafer are shown in Fig. 7D. The size of the detected Micro-LED wafer is 4 inches, and the size of a single pixel is 40 μm × 20 μm. Although NC-EL detection offers distinct advantages in acquiring Known Good Die (KGD) data, it currently lacks the capability to batch-measure typical electrical parameters such as *V*_F_, *I*_F_, and *I*_r_ and can only obtain the AC *I*–*V* curves of Micro-LED chips. In the future, the integration of artificial intelligence (AI) methodologies with large-volume data analytics is expected to enable the derivation of these electrical parameters via NC-EL.

In recent years, some companies have industrialized and sold finished products of NC-EL detection equipment, and NC-EL detection is gradually becoming a mature Micro-LED large-scale detection technology. In 2019, Tesoro Scientific proposed a detecting device and manufacturing method for LEDs [56]. By using a field plate and injecting current through a displacement current coupling device, the LED array is detected. In addition, Top Engineering of South Korea announced the TNCEL-W Micro-LED noncontact detection equipment in 2021, which can batch detect Micro-LED chips smaller than 50 μm in the wafer process stage. The equipment applies both electrical and optical detection methods, which both do not require direct contact with Micro-LEDs.

#### PL detection

**Fig. 8.**
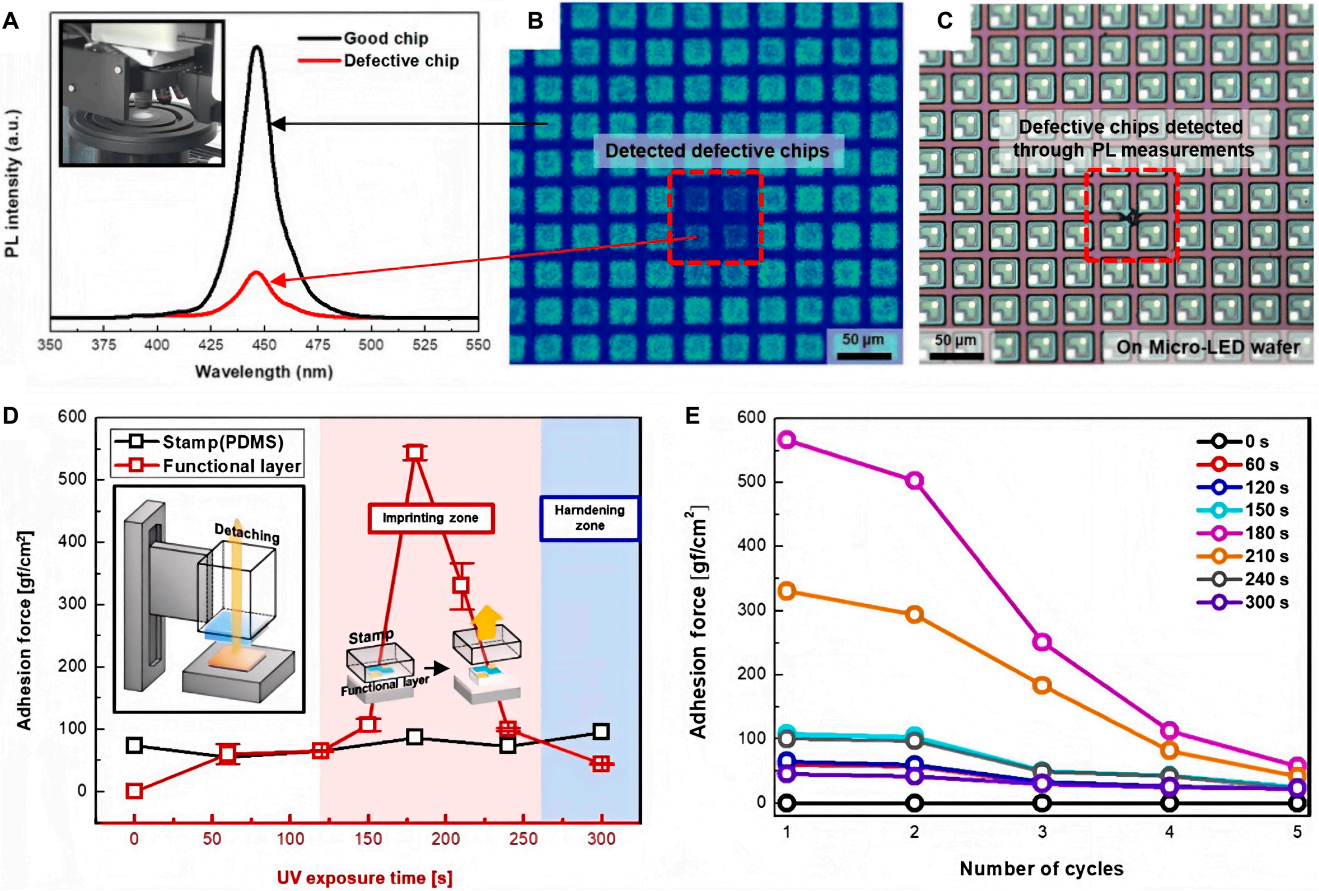
(A) Comparison of PL intensity between good and defective chips detected by micro-pulsed laser [60]. (B) PL intensity image of the detected Micro-LED after micro-pulsed laser irradiation [60]. (C) Optical microscopy image of defective chips on a real Micro-LED wafer found using a PL intensity image [60]. (D) Adhesion force between the stamp and the functional layer as a function of UV exposure time [60]. (E) Repeated adhesion force of the functional layer as a function of UV exposure time, measured from 0 to 300 s at 60-s intervals [60]. (A to E) Reprinted with permission from [60], under the CC BY 4.0 license.

PL detection is a commonly used detection method in Micro-LED detection. In addition to using conventional UV light to excite Micro-LED chips, high focusing micro-pulse lasers can also induce PL in Micro-LEDs. In 2018, Park et al. [60] analyzed the performance of a blue Micro-LED array using a 375-nm wavelength micro-pulse laser. They use lasers to inject photons from outside the Micro-LED chip, and the energy of the photons causes the electrons and holes inside the Micro-LED to undergo radiative recombination, resulting in 450-nm blue light. From the PL intensity graph, it can be observed that the PL intensity of defective products is significantly lower than that of normal chips (as shown in Fig. 8A). It can also be clearly observed from the PL image of the Micro-LED array obtained by the camera that the luminescence intensity of defective products is significantly lower than that of the surrounding normal chips. However, the Micro-LED array under natural light showed no defects or damage (as shown in Fig. 8B and C). Therefore, the results of PL detection can be used to determine the quality of Micro-LEDs, in order to screen and replace defective Micro-LED chips in the subsequent process. The laser spot used can be adjusted to less than 2 μm. Therefore, their method is expected to be used for PL detection and analysis of individual Micro-LEDs. In addition, this method is combined with photosensitive materials. After PL detection of Micro-LEDs, if defective chips are found, they can be quickly screened and replaced, improving the process efficiency of Micro-LEDs. Figure 8D shows the adhesion of photosensitive materials at different UV exposure times. The different adhesive forces correspond to different processes. However, after multiple cycles, the adhesive strength of the photosensitive material will significantly decrease. To avoid the decrease in yield caused by the failure of photosensitive materials, Park et al. also summarized the service life of photosensitive materials under different UV exposure times, as shown in Fig. 8E. These data not only reflect the specific lifespan of the seal under UV pulses but also can be combined with detection processes to accurately screen out defective chips after detection.

PL detection has significant advantages in improving the efficiency of Micro-LED processes, and finding the relationship between PL detection results and *I*–*V* characteristic curves may further enhance efficiency. In 2024, Hong et al. [61] analyzed the correlation between PL spectra and *I*–*V* characteristic curves of different LEDs, and studied the feasibility of predicting *I*–*V* characteristic curves through PL detection results. Firstly, they changed the excitation light power and detected the PL spectral characteristics of different samples. Subsequently, they conducted traditional EL detecting on these samples. The detect results indicate that the *I*–*V* characteristic curve results show significant differences between different samples, which is consistent with the PL detection results. At the same time, they established corresponding carrier transport models and fitted *I*–*V* characteristic curves to obtain ideal factors for different samples. The results indicate that samples with lower PL intensity exhibit a larger ideal factor in the *I*–*V* curve. On the contrary, for samples with high PL intensity, the ideal factor obtained is relatively small, which is close to 2. From the transmission electron microscopy image of the sample, significant enrichment of In and the presence of V-pit on the sidewall can be observed. Both caused the yellow emission band and excessive blue shift of peak wavelength in the PL spectrum, respectively.

In 2021, Behrman et al. [62] proposed the idea of using PL imaging and CL imaging instead of EL detection to achieve nondestructive detection. They found that for the same multi-pixel LED sample, when excited with short-wavelength light, all pixels emit light, but not all pixels are illuminated when using CL imaging. The explanation for this is that the entire process of PL does not involve carrier transport throughout the device, and is therefore not affected by various short-circuit defects generated by LED pixels after etching. In order to ensure the accuracy of the EL detection image, a series of processing was carried out on the LED pixels and a customized PCB board was used for detection. The experiment found that 71.75% of pixels emit light in both CL and EL images, indicating that CL images can fit EL detection results to a certain extent, but the fit still needs to be further improved.

#### AOI

AOI is a commonly used LED defect detection technology on the market today. With the development of convolutional neural networks (CNNs), the efficiency and accuracy of AOI systems are also increasing, and different algorithms can adapt to different detection requirements.

In 2021, Weng et al. [69] used scale invariant feature transform (SIFT) and Harris Laplace method to search for feature regions on LED chip patterns and analyze the lithography patterns of LEDs. To overcome the defect detection errors caused by displacement and scale changes in photolithography patterns, an adaptive template was established under actual conditions. In order to improve the positioning accuracy of the adaptive template, a fast chip integrity screening algorithm was introduced before positioning, which improved the efficiency of system judgment. Comparing this method with the template comparison method, it was verified that the adaptive template can overcome the problem of poor fitting of the standard template, emphasizing that this method can improve product detection accuracy and industrial production efficiency. The results indicate that the adaptive template method can achieve a detection accuracy of 98.63%. This method not only has high accuracy but also can locate the specific characteristics of different sizes of LEDs with the same structure and the appearance defects of LED chips.

**Fig. 9.**
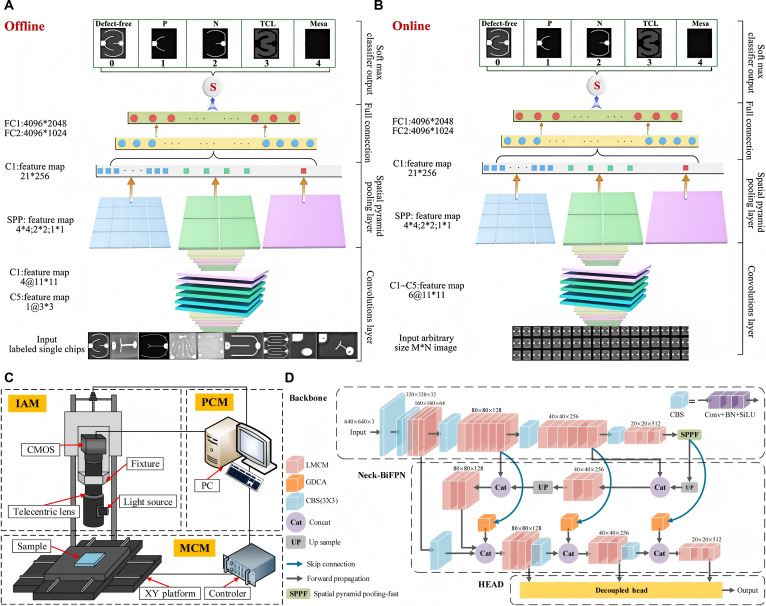
(A) Offline training SPP-net model [71]. (B) Online detecting SPP-net model [71]. (C) Schematic diagram of the microscopic vision-based detection system [74]. (D) Structure of the proposed LBG-YOLO [74]. (A and B) Reprinted with permission from [71]. Copyright 2021, Elsevier. (C and D) Reprinted with permission from [74]. Copyright 2024, Elsevier.

In 2021, Shu et al. [71] proposed a parallel deep convolution model for surface quality detection of LED chips, called parallel spatial pyramid pooling network (PSPP net). The advantage of this model is that it can greatly reduce the image capacity obtained by the camera, so as to ensure that an image covers a sufficient number of LED chips while maintaining sufficient clarity on the basis of high resolution. The detection process is shown in Fig. 9A and B. From the microscopic images, it can be clearly observed that there are defective LED chips in the LED array. They detected LED chip images with different brightness, noise, and image rotation angles and then demonstrated their predictive performance using receiver operating characteristic (ROC) curves. The area under the curve (AUC) is defined as the area under the ROC curve. The results indicate that the trained model not only has good robustness to changes in light and rotation of the detected LED wafer (i.e., changes in light and rotation of the detected LED wafer do not have a significant impact on detection accuracy) but also has a certain ability to resist noise interference. Finally, by comparing different target recognition algorithms, it was verified that the model has satisfactory accuracy, speed, and universality, significantly better than traditional algorithms.

In 2024, Chen et al. [74] constructed a micro-vision-based automatic detection system (MVDS) to capture images of Micro-LED chips and proposed a deep learning algorithm called LBG-YOLO. The schematic diagram of the detection system and the structure of LBG-YOLO are shown in Fig. 9C and D, respectively. This deep learning algorithm is based on YOLOv8s and combines lightweight multi-scale convolution module (LMCM), bidirectional feature pyramid network (BiFPN), and global dependency coordinate attention (GDCA) mechanism. These structures aim to improve the feature extraction and fusion capabilities of the algorithm, enabling it to fuse features more effectively, reduce the size of the network, fully utilize location information, and more accurately locate targets. The LBG-YOLO has the strongest defect identification ability among the listed models. Experiments on the public dataset VOC2012 have proven the validity of this structure, with the mean accuracy precision (mAP) of 90.8%.

#### Microscopic defect detection

Microscopic defects have a small proportion of volume in a single LED pixel for conventional-sized LEDs (millimeter level) and have almost no impact on the photoelectric characteristics of the LED pixel. At this point, microscopic defects can be ignored. However, the size of Micro-LEDs is extremely small (less than 50 μm), and the proportion of micro-defects in the volume of a single pixel increases. If conventional processes are used to manufacture Micro-LEDs, the micro-defects will definitely seriously affect the optoelectronic properties of Micro-LEDs. Therefore, using reasonable detection methods to detect and analyze micro-defects in Micro-LEDs, and then improving the preparation process of Micro-LEDs, can further promote the industrialization process of Micro-LEDs.

**Fig. 10.**
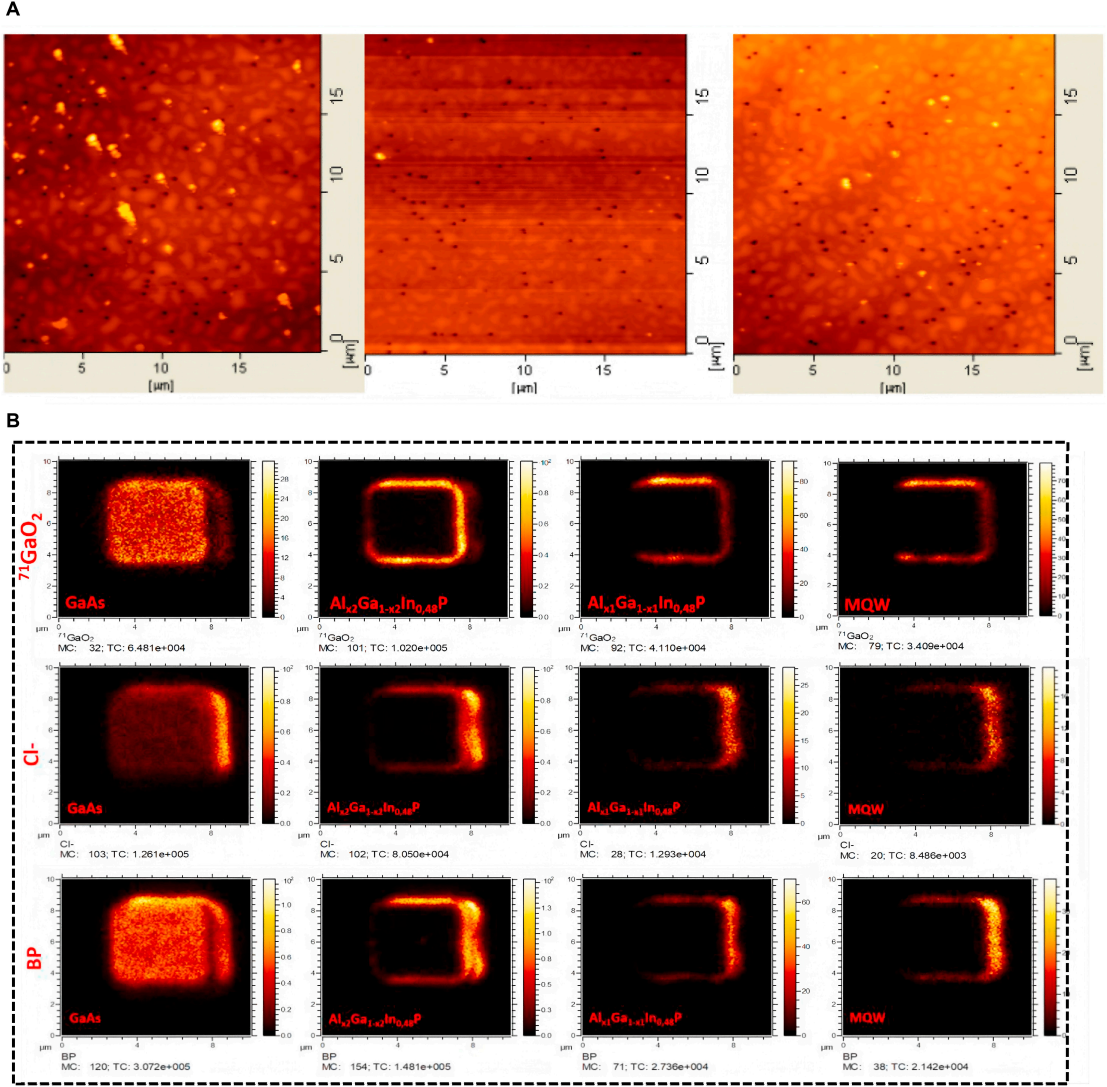
(A) AFM surface scans of the 520-, 540-, and 550-nm wafers (left to right) [76]. (B) TOF-SIMS maps of chlorine and boron [80]. (A) Reprinted with permission from [76], under the CC BY 4.0 license. (B) Reprinted with permission from [80]. Copyright 2021, Elsevier.

Scanning electron microscopy (SEM) and atomic force microscopy (AFM) are commonly used surface defect observation methods for conventional-sized LEDs. The detection results of AFM are shown in Fig. 10A. They are commonly used for surface morphology analysis and quality control of conventional-sized LEDs. SEM can provide high-resolution surface images, while AFM can accurately 3D image the microstructure of material surfaces, which is widely used in the manufacturing and detection of conventional LEDs. However, with the emergence of Micro-LEDs, the size has been reduced to the micrometer or even nanometer level, and the accuracy of SEM and AFM may not meet the higher requirements for fine structure analysis in some cases, especially in terms of accuracy and resolution at small scales such as pixel spacing and defect detection. Therefore, the development and manufacturing of Micro-LEDs require more precise characterization techniques to meet their higher precision requirements.

In the early days, the sidewall defects of μLED or Micro-LED were usually determined based on the measurement results of current–voltage–luminescence (*I*–*V*–*L*) measurements [77–79], which could not accurately obtain the actual affected area of sidewall defects during the MESA stage. In 2021, Boussadi et al. [80] proposed an alternative method that couples optical characterization techniques with time-of-flight secondary ion mass spectrometry (TOF-SIMS). This method is used to analyze sidewall defects on AlGaInP of different sizes formed after reactive ion etching (RIE) with BCl_3_ ion beam. The experimental results indicate that for 6 × 6 μm^2^ pixels, the uniformity of light emission is largely affected by sidewall defects. Based on the emission efficiency map derived from temperature-dependent CL measurements, we estimate that 84.5% of the efficiency in 6 × 6 μm^2^ pixels is lower than the center. Due to nonradiative recombination, the carrier lifetime extracted from TRPL measurements on larger pixels gradually decreases at a distance of 3 μm from the sidewall. TOF-SIMS analysis revealed residual boron and chlorine on the pixel surface and sidewalls after BCl_3_ etching. The detect results are shown in Fig. 10B. This detection method can reflect the cause of the defects on the side wall after etching, which has a strong guiding role for Micro-LED technology.

### COC and packaging detection

Post-packaging detection of Micro-LEDs is a critical step in ensuring the performance, reliability, and overall quality of Micro-LED displays. Given the extremely small size and high integration of Micro-LEDs, even minor defects or inconsistencies during the packaging process can impact display performance, lifespan, and brightness. Therefore, precise post-packaging detection is essential for promptly identifying issues such as solder joint defects, inconsistent optoelectronic performance, color deviation, and other potential faults, ensuring that each Micro-LED module meets stringent quality standards [81–97]. Additionally, this detection process helps optimize production workflows, reduce scrap rates, and improve overall production efficiency, which is vital for the large-scale adoption of Micro-LEDs. Particularly after packaging, it is crucial to check for any open circuits in the Micro-LEDs, as this can significantly affect the functionality of the entire display.

**Fig. 11.**
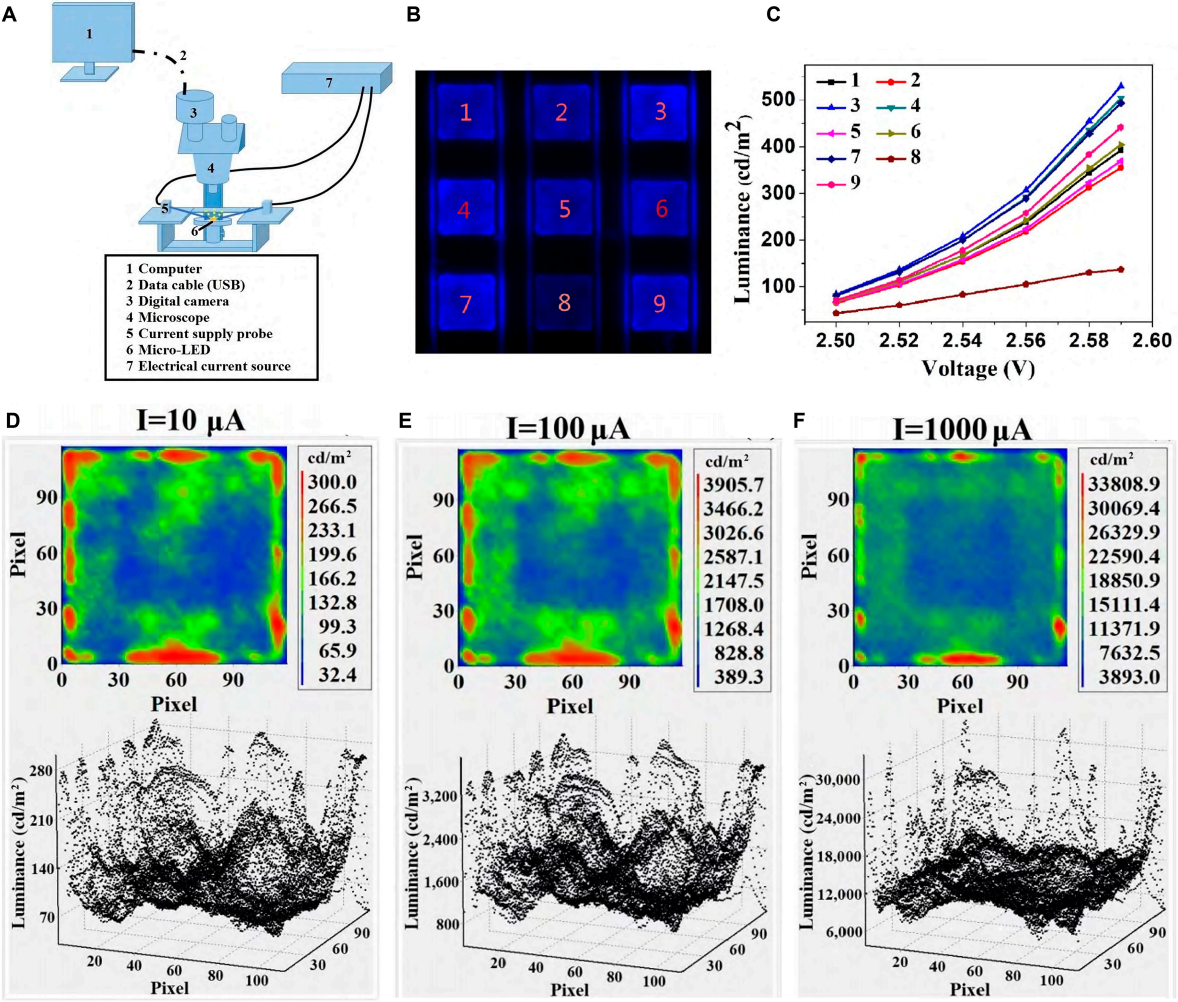
(A) Schematic diagram of microscopic luminance measurement system for the Micro-LED array [81]. (B) Certain chips with brightness differences on the Micro-LED array [81]. (C) Average luminance of chips in (B) under different voltages [81]. (D to F) Pseudo-color map and 3D distribution of the luminance from a single Micro-LED chip, measured at current levels of 10, 100, and 1,000 μA [81]. (A to F) Reprinted with permission from [81], under the CC BY 4.0 license.

Capturing the micro-surface brightness of Micro-LED arrays and studying their micro-characteristics is of great significance for the optical and electrical parameters of Micro-LEDs. In 2018, Zheng et al. [81] proposed a detection method that can measure the surface brightness distribution of the entire Micro-LED array and even a single Micro-LED chip. The diagram of the detection system is shown in Fig. 11A. Before detection, the Micro-LED array needs to be transferred to a specially designed circuit. This circuit can be selected through row and column electrodes, thus meeting the requirements of lighting up a single Micro-LED chip and multiple Micro-LED chips simultaneously and can be detected using a measurement system in 2 different situations. The obtained partial Micro-LED array images and corresponding *I*–*V* characteristic curves of Micro-LED chips are shown in Fig. 11B and C, respectively. In addition, the camera system will generate a pseudo-color image through an algorithm system after capturing images of the glowing Micro-LED array. From the pseudo-color image, it can be seen that as the current density increases, the uniformity of the surface brightness of Micro-LED increases, as shown in Fig. 11D to F. The final detection results show that the brightness values obtained by the detection system have a maximum error of less than 8% compared to traditional detection methods, proving that the method is accurate. This method can accurately characterize the brightness uniformity of a single LED chip in terms of detection and can also simultaneously characterize the brightness uniformity of multiple LED chips. The drawback is that the cost of producing electrodes with multiple rows and columns is relatively high, and these transitional electrodes need to be removed in the later stage.

Micro-electromechanical systems (MEMS) technology involves the design, processing, manufacturing, measurement, and control of micro/nanomaterials. It enables the integration of mechanical components, optical systems, actuators, and electronic control systems into a unified micro-system. One notable application of MEMS technology is the MEMS switch. A MEMS switch is a 3-terminal device. By adjusting the voltage of the gate, the on/off state of the source and drain can be controlled. By properly modifying and utilizing MEMS switches, a Micro-LED detection device can be obtained. In 2021, BOE company proposed a flexible probe based on MEMS switch principle [83]. It can control the state of the cantilever by adjusting the gate voltage. When the gate voltage is 0 V, the cantilever remains stationary due to the resistance of the spring. When a voltage of 10 to 20 V is applied to the gate, the cantilever overcomes the resistance of the spring, causing the source and drain to come into contact and conduct. By adjusting the magnitude of the gate voltage and the elasticity coefficient of the spring, the weight of the contact between the cantilever terminal and Micro-LED can be adjusted to avoid stress concentration.

**Fig. 12.**
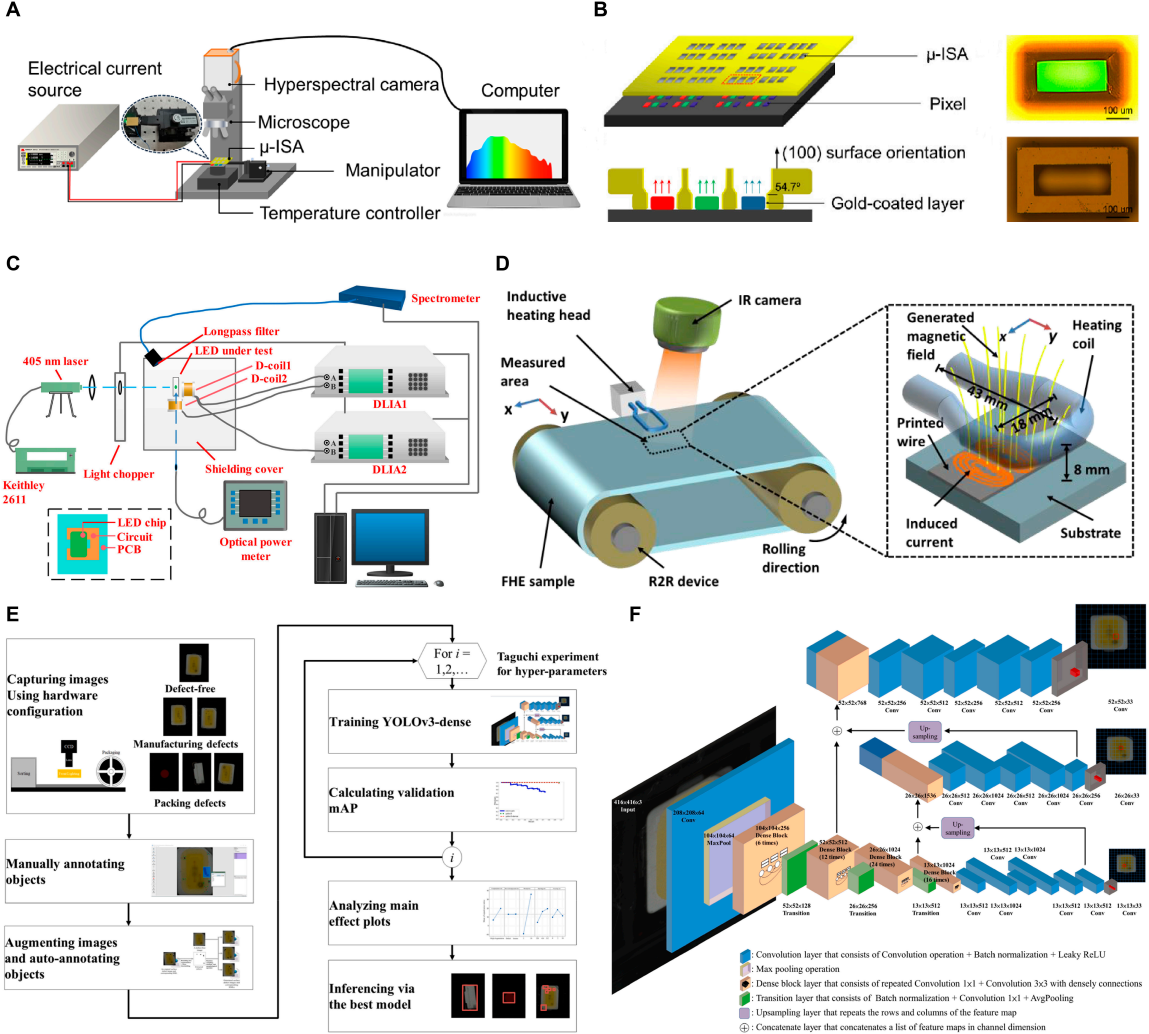
(A) Schematic of the measurement system [85]. (B) μ-ISA and Micro-LED array transferred onto carrier [85]. (C) Schematic diagram of the noncontact detection system, with an inset showing a schematic diagram of a Mini-LED on a printed circuit board (PCB) [86]. (D) Schematic of the noncontact inductive heating-based R2R characterization system [87]. (E) Research process of SMD LED chip defect detection [88]. (F) YOLOv3-dense network structure [88]. (A and B) Reprinted with permission from [85]. Copyright 2023, IEEE. (C) Reprinted with permission from [86]. Copyright 2024, IEEE. (D) Reprinted with permission from [87]. Copyright 2020, IEEE. (E and F) Reprinted with permission from [88]. Copyright 2021, Elsevier.

As is well known, LEDs are current mode devices, and their luminous intensity is very weak under low driving current, which increases the difficulty of detection. Therefore, it is very important to solve the difficulty of completely capturing weak signals under small driving currents while avoiding optical crosstalk from adjacent chips. In 2023, Li et al. [85] proposed an efficient measurement method. This approach integrates micro-hyperspectral imaging (μ-HSI) with a silicon-based integrated sphere array (μ-ISA). A truncated inverted pyramid microcavity coated with a gold reflective layer was fabricated within the μ-ISA, enabling effective collection of the total optical power from a Micro-LED array while minimizing optical crosstalk from neighboring chips. Schematic diagrams of the detection system and the μ-ISA are provided in Fig. 12A and B, respectively. A set of single RGB Mini-LEDs and Mini/Micro-LED arrays was characterized using both the proposed method and conventional spectrometer-based measurements for comparison. In contrast, when the μ-ISA is employed, such interference is substantially suppressed or eliminated, as indicated by the solid curves. The maximum deviation in measurement accuracy for the proposed system remains below 3.67%. Experimental results confirm that the μ-ISA effectively mitigates interpixel optical crosstalk and facilitates high-sensitivity batch detection. Nevertheless, the μ-ISA fabricated via this process is tailored to specific Micro-LED array layouts, imposing strict alignment requirements and limiting general applicability across different array configurations.

From the analysis earlier in this article, it can be seen that the existing PL detection has the problem of artificially high yield rate. Moreover, PL detection cannot detect the critical reverse saturation current. There is an urgent need to develop a method to detect the optoelectronic characteristics of chips before or during the packaging process, in order to effectively ensure product quality. In 2023, based on the electromagnetic coupling theory and photovoltaic effect, Zhong et al. [86] proposed a noncontact photoelectric joint detection method. The schematic diagram of the detection system is shown in Fig. 12C. This method utilizes a short-wave laser to excite the photocurrent of Mini-LED in a closed loop, obtains differential induction signals by using 2 orthogonal coils, and detects them through a lock-in amplifier. The proposed method can be used to derive the key optoelectronic parameters of Mini-LEDs, especially the *I*–*V* relationship, *I*_r_, peak emission wavelength, and other key parameters. A set of green Mini-LED samples was evaluated, and the experimental data aligned with theoretical predictions, confirming the viability of this approach for spectral analysis, Mini-LED KGD identification, and electrical performance detection. It can be observed that there is almost no difference between the EL spectrum of sample 1 and the PL spectrum at different powers, which proves the reliability of the optical parameters measured by the system. In addition, they also detected the *I*_r_ of 4 normal samples under different optical powers with a fixed reverse bias (5 V). The experimental results show that the minimum estimation error of sample 4 is about 2.83%, and the average error of the 4 samples is about 18.07%, indicating that the system based on LED noncontact evaluation is feasible.

Eddy current heating (ECH) can induce current in silver wires prepared on special systems, causing multiple LED chips mounted on the silver wires to simultaneously produce EL and improve detection efficiency. Remes and Fabritius [87] proposed a new method for detecting the luminescence of LED chips. The system uses a roll to roll (R2R) method to transmit new LED products and continuously scans the sample with a camera, as shown in Fig. 12D. In addition, the system uses ECH to induce current in the preset silver wire to light up the LED chip and combines synchronous thermal imaging to obtain the light emission of the LED chip. The first sample is an intermediate product with only silver wires printed on the roll-to-roll system, without LED chips installed on its surface. The other sample is equipped with LED chips on the basis of the previous sample. If the quality of the LED chip is qualified, it will be illuminated by the induced current generated in the preset silver wire by the system after ECH, thereby producing greater intensity on the infrared thermal image. The images indicate that the sample loaded with LED chips has a higher intensity in the thermal image, reflecting the advantage of the system in batch detection of LED chips. However, this detection system has certain drawbacks in the detection of LED chip defects, namely, the significant temperature distribution differences between each LED chip. That is to say, although both samples are loaded with LED chips, their infrared image intensity when emitting light is also different. Without further quantitative analysis and improvement of the detection system, it is impossible to determine whether LED chips have defects solely based on the intensity of thermal imaging. For Micro-LED chips, this detection system will also encounter the same problems as mentioned above.

In 2021, Chen and Tsai [88] improved YOLOv3 and proposed the YOLOv3 dense model for defect detection of surface mount device LEDs (SMD LEDs). Its essence is to use Dense-net instead of Darknet-53. The research process of SMD LED chip defect detection and the YOLOv3-dense network structure are shown in Fig. 12E and F, respectively. The status of SMD LEDs can be divided into 6 categories: no defects, missing components, incorrect placement, polarity reversal during tape application, missing gold wires, and surface defects. The 6 states consist of approximately 100 images each. The original images consist of 726 images, and in order to enrich the image features, data augmentation methods were used to generate pseudo-images for each category. Approximately 400 pseudo-images were generated for each category, for a total of 2,433 images, to ensure that YOLOv3 dense is fully trained. As shown in the precision–recall (PR) curves, YOLOv3 dense has demonstrated high accuracy in detecting performance across 6 categories, with an average detecting accuracy of 95.28%. With such precise classification of defect categories, the average detecting accuracy can reach over 95%, indicating that this scheme is effective and reliable.

In addition, Wen et al. [89] proposed a noncontact detection method using light excitation and light detection. By obtaining the PL spectrum generated by the PN junction of the light excited LED, and establishing the exact relationship between the PL and EL effects of the LED, as well as the corresponding relationship between the emission spectrum characteristics of the LED and its electrical characteristics, the PL spectrum is analyzed and processed to determine the electrical characteristic parameters of the LED. Using *T*_j_ as a “bridge” between the PL spectrum and EL spectrum of the LED, the PL spectrum is used to make *T*_j_ of the PN junction of the detected LED consistent with the EL spectrum, and the EL spectrum at the same *T*_j_ is equivalent to the PL spectrum. It can be observed that in the case of small injection, the fitting degree of this method is high, but as the injection current increases, the estimated value is slightly smaller than the actual value. This is due to the influence of the internal resistance of the LED itself on the electrical characteristics under different injection methods. This method uses *T*_j_ to link the PL spectrum with the EL spectrum, equating the PL spectrum and EL spectrum of LEDs at the same *T*_j_. It can effectively predict the emission spectrum of LEDs during normal operation, but cannot achieve the goal of screening bad pixels in LED chip arrays.

Simultaneously detecting Micro-LED chips during mass transfer can significantly shorten the manufacturing process time of Micro-LEDs. Zhang et al. [92] proposed a detection method that balances rough detection of Micro-LED chips and transfer of Micro-LED chips. First, the Micro-LED chip is fabricated on a transient substrate coated with UV glue, and then it is detected using a detection substrate with a photosensitive conductive layer. The detection step is to bond the photosensitive electrode layer on the detection substrate with the epitaxial layer of the Micro-LED chip on the transient substrate, and then use visible light irradiation. The adhesive force of the photosensitive electrode layer will be greater than that of the UV glue on the transient substrate. At this time, only normal Micro-LEDs can be cured and bonded with the photosensitive conductive layer. Micro-LED chips with problems after epitaxy and etching cannot be bonded by the photosensitive conductive layer. The bonded chip can be transferred to the display device. This method combines early quality detecting and transfer steps for Micro-LEDs before transfer, but it is unknown whether the transferred Micro-LED chip can function properly.

### Panel detection

In addition to surface defects on the device, there may be some pixel breaks in the Micro-LED display screen after transfer to the circuit. To reduce costs, a nondestructive and rapid detection method is needed to obtain internal defects and wire connections of Micro-LEDs [98–109].

**Fig. 13.**
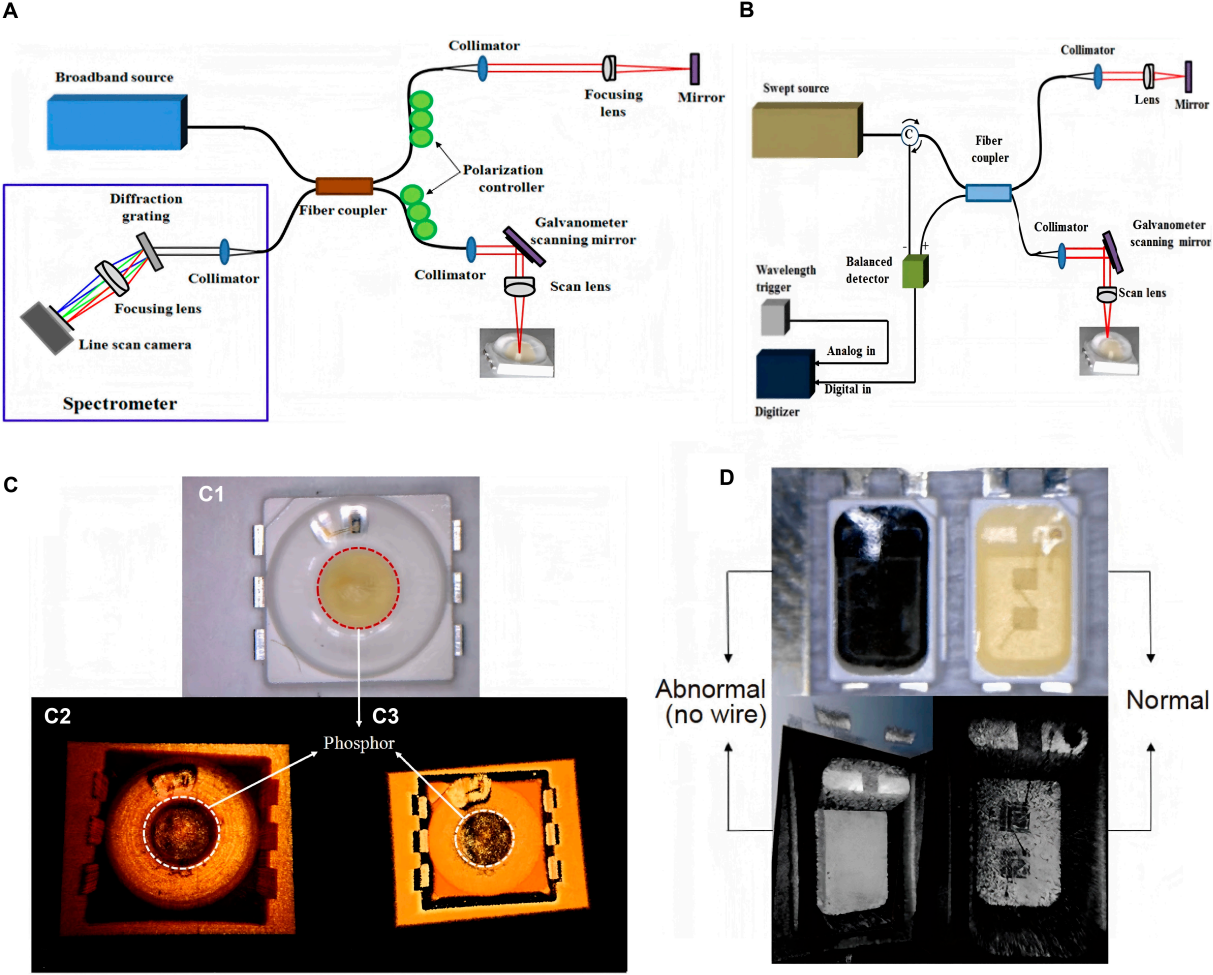
(A) Schematic diagram of the SD-OCT system. (B) Schematic diagram of the SS-OCT system. (C) Composite image showing microscope top view (top), 850-nm SD-OCT 3D top view (lower left), and 1,310-nm SS-OCT 3D top view (lower right). (D) Comparison of digital microscope image (top) and OCT image (lower) of abnormal (no wire) LEDs and normal LEDs. (A to D) Reprinted with permission from [99], under the CC BY 4.0 license.

As early as 1991, optical coherence tomography (OCT) was proposed [98]. In 2012, Cho et al. [99] proposed a detection method for nondestructive acquisition of internal information of Micro-LEDs using OCT, including spectral domain OCT (SD-OCT) and scanning source OCT (SS-OCT). The schematic diagram of the detection system is shown in Fig. 13A and B, respectively. They compared SD-OCT and SS-OCT images of the same sample to study their advantages. From Fig. 13C, it can be observed that the distribution of fluorescent materials is clearer in the SD-OCT at 850-nm wavelength, while the state of wire connections is clearer in the SS-OCT image at 1,310-nm wavelength. By combining the 2 OCT images obtained, the bad pixels of the LED screen can be clearly captured. As shown in Fig. 13D, it can be clearly seen from the OCT image that there is a problem of missing wires in the abnormal LED chip. However, the sample detected by this method is LED, and it can be found from the experimental data that applying this method to Micro-LED detection will result in insufficient clarity. In the future, this method is expected to be applied to the detection of downstream Micro-LED products by improving equipment accuracy or enhancing the structure of detection systems.

**Fig. 14.**
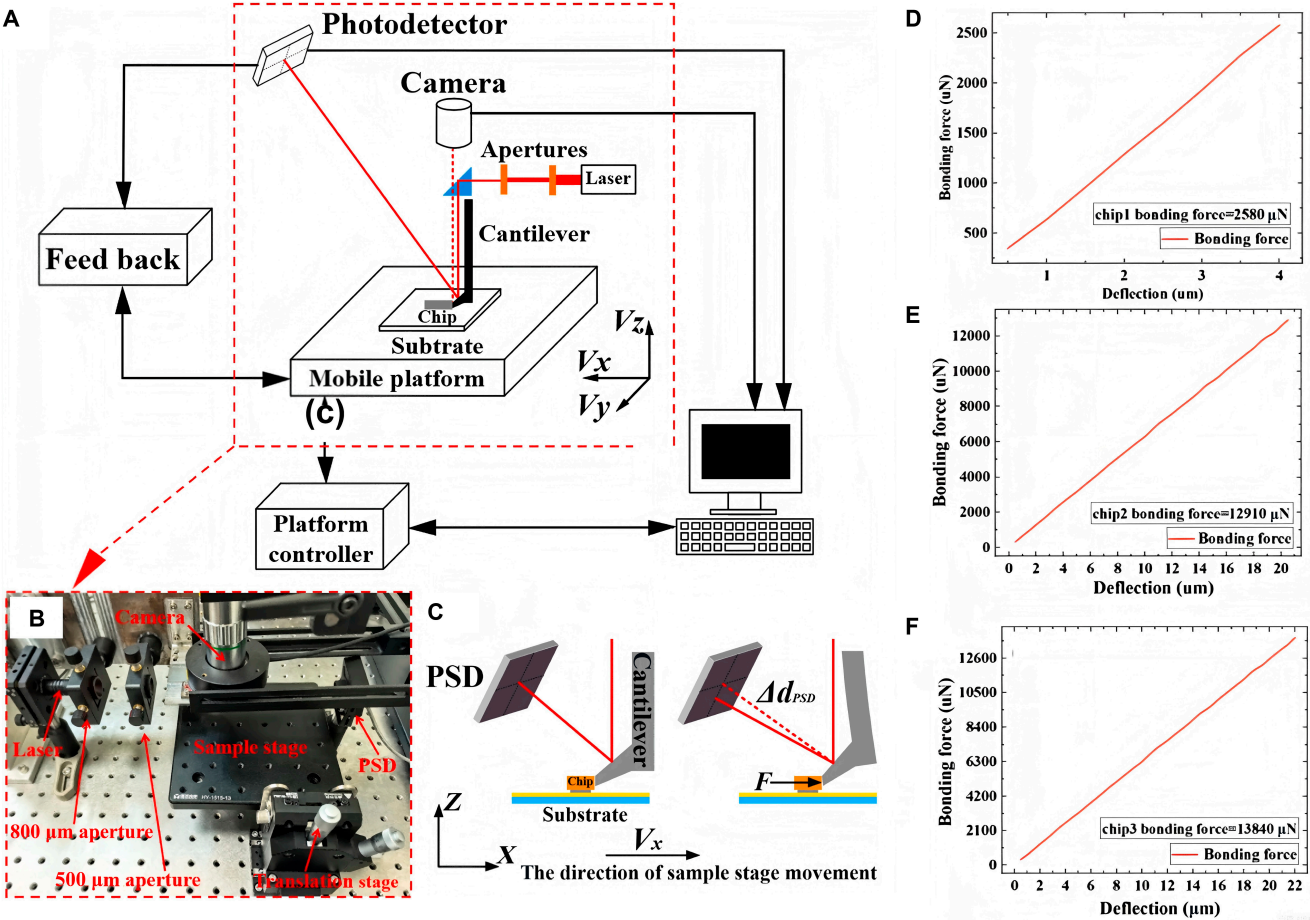
(A) Schematic diagram of the detection system. (B) Physical diagram of the core part of the detection system. (C) Detection principle. (D to F) Illustrations of bonding force profiles of the 3 different Micro-LED chips. (A to F) Reprinted with permission from [101]. Copyright 2025, Elsevier.

The adhesion strength of Micro-LED chips on the panel has a significant impact on the stability and imaging quality of the display panel. Therefore, chips can be screened by measuring the bonding force of Micro-LED chips on the panel, removing defective chips with insufficient bonding force and replacing them. In 2025, Qiao et al. proposed a bonding force detection system panel-level Micro-LED based on the principle of optical lever. The system schematic is shown in Fig. 14A, with the red dashed box indicating the core part of the detection system. This part is configured on an optical shock-resistant platform to ensure stability during detecting, as shown in Fig. 14B. During the detection process, the horizontally incident laser is vertically incident on the reflective surface of the contact head through an optical guidance module, and then received by a position sensitive detector (PSD). When the contact head comes into contact with the Micro-LED chip, it causes deformation of the lever cantilever connected to the contact head, and the angle between the reflective surface on the contact head and the *z* axis also changes, resulting in a change in the position of the laser spot received by the PSD, as shown in Fig. 14C. This detection method reflects the bonding force of Micro-LED chips on the panel by the degree of change in the spot position and is characterized by a precalibrated force–cantilever deformation curve, as shown in Fig. 14D to F. This scheme, which characterizes the quality of panel-level Micro-LED chips using the magnitude of bonding force, has high accuracy and is easy to establish standards. However, limited by the size of the contact head, the spacing between Micro-LED chips cannot be too small; otherwise, it will affect the normal operation of the contact head, which is not suitable for high pixels per inch (PPI) Micro-LED display panels.

## Conclusion and Prospect

**Fig. 15.**
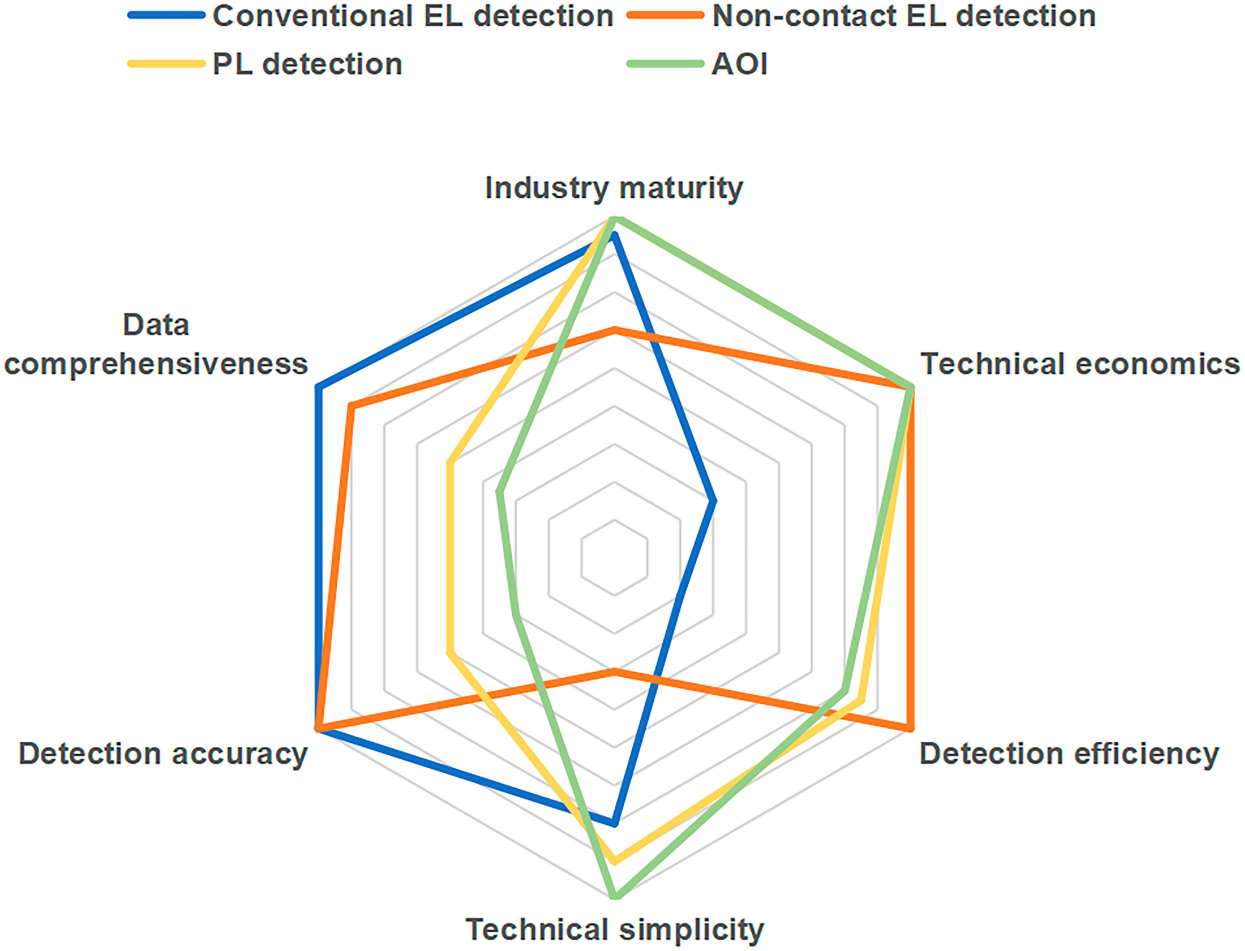
Comparison of LED detection technologies.

From the above introduction of the detection methods corresponding to each step in the Micro-LED process flow, it can be seen that there are usually multiple techniques to choose from for the same process step. However, some of these technologies are still in the laboratory stage and are not yet suitable for large-scale production lines. Therefore, based on the practical requirements of Micro-LED industrialization and the challenges in the current manufacturing process, we have identified detection technologies that are already applicable to production lines or show great potential for future adoption. In addition, we have analyzed the advantages and disadvantages of these detection techniques from multiple dimensions, including industry maturity, techno-economic feasibility, detection efficiency, technical simplicity, detection accuracy, and data comprehensiveness, based on an analysis of the strengths and weaknesses of various detection methods reported in the literature, combined with the practical requirements of Micro-LED industrialization, and in light of the current challenges and needs in Micro-LED manufacturing processes. The results are presented in Fig. 15. As the most established inspection method, conventional EL detection benefits from a prolonged period of research and refinement, resulting in high technical maturity. However, the necessity for physical contact with the chips via probes, coupled with complex equipment and its typical application in sampling inspections, leads to poor technical economics and extremely low detection efficiency. Leveraging mature semiconductor inspection equipment such as semi-automatic probe stations, its system operation is relatively straightforward. Crucially, it can simultaneously acquire both optical and electrical parameters of Micro-LED chips under conditions that closely mimic actual device operation. Consequently, it achieves the highest level of both detection accuracy and data comprehensiveness, making it the benchmark for in-depth failure analysis and performance validation. NC-EL detection excites EL without physical contact. Proposed relatively recently, its novelty results in a moderate level of technical maturity. By eliminating direct contact with the Micro-LED chips, it reduces physical damage and lowers overall costs, thereby enhancing its technical economics. Furthermore, its inherent capability for parallel inspection of thousands of chips simultaneously yields extremely high detection efficiency. However, the sophisticated system requirements and complex implementation detract from its technical simplicity. While its detection accuracy approaches that of conventional EL methods and data comprehensiveness is relatively high, it may not capture certain subtle details accessible only through physical contact-based conventional EL inspection. PL detection is already widely adopted for semiconductor material characterization and exhibits the highest level of industrial maturity. The primary consumable-related cost involves the degradation of the UV light source over its lifespan, and the equipment itself is relatively inexpensive, resulting in excellent technical economics. This technique also supports the simultaneous inspection of multiple chips, affording relatively high detection efficiency. It allows for rapid switching of the inspection field of view, contributing to operational simplicity. Nevertheless, as optical excitation cannot fully replicate the effects of electrical current injection, the obtained data may exhibit discrepancies compared to EL measurements, limiting its detection accuracy. Moreover, its inability to acquire electrical parameters of the Micro-LED chips restricts its data comprehensiveness. AOI is mature, is widely adopted, and offers controllable equipment costs. It enables the inspection of the surface morphology of numerous Micro-LEDs within a single field of view, resulting in fast inspection speeds. The core of this technique lies in the CNN algorithms tailored to the specific Micro-LED chips under test, ensuring a high degree of automation and low system complexity. However, as AOI primarily relies on surface image analysis, it cannot directly probe internal electrical performance or subsurface defects. Consequently, its detection accuracy and data comprehensiveness are comparatively low, rendering it suitable primarily for rapid, high-throughput preliminary screening.

With the continuous advancement of Micro-LED display technology, high resolution is destined to become core features of future displays. At the same time, as the manufacturing processes of display devices continue to improve, the bottleneck in technological development has gradually shifted from chip fabrication to subsequent stages represented by quality detection. Taking a 6-inch wafer with 5-μm LED chips (~5,000 pixels per inch in display panel) as an example, detecting tens of millions of Micro-LED chips on such a wafer is challenging. In this case, conventional EL detection has become ineffective at this scale, while PL detection suffers from limited accuracy due to low signal fidelity. Although AOI is an option, its detection precision is insufficient for high-yield requirements, and identifying defects in such micrometer-scale devices pushes optical systems to their fundamental limits. Consequently, NC-EL detection is poised to become the gold standard for next-generation detection. The advantage of NC-EL detection is that it can avoid the problem of high missed detection rate and accurately obtain the EL performance of Micro-LED chips. At the same time, the detection efficiency can be comparable to PL detection and AOI, which can effectively shorten the process time. In addition, NC-EL detection can achieve batch detection of Micro-LED chips (the number of detection depends on the area of the detection probe) and will not apply any external force to the Micro-LED chips, eliminating additional damage to the chips caused by detection. With the rapid development of Micro-LEDs in large screens and high-definition displays, contactless and fast electrical detection solutions are the trend.
